# Chromosome-scale genome, together with transcriptome and metabolome, provides insights into the evolution and anthocyanin biosynthesis of *Rubus rosaefolius* Sm. (Rosaceae)

**DOI:** 10.1093/hr/uhae064

**Published:** 2024-03-02

**Authors:** Yunsheng Wang, Jiyuan Guan, Qunying Zhang

**Affiliations:** School of Health and Life Science, Kaili University, Kaili city, Guizhou Province 566011, China; Botanic Garden of Guizhou Province, Guiyang city, Guizhou Province 550081, China; Botanic Garden of Guizhou Province, Guiyang city, Guizhou Province 550081, China

## Abstract

*Rubus rosaefolius* is a kind of red raspberry possessing high nutritional and pharmaceutical value. Here we present a chromosome-level draft genome of *R. rosaefolius.* Of the total 131 assembled scaffolds, 70 with a total size of 219.02 Mb, accounting for 99.33% of the estimated genome size, were anchored to seven pseudochromosomes. We traced a whole-genome duplication (WGD) event shared among members of the Rosaceae family, from which were derived 5090 currently detectable duplicated gene pairs (dgps). Of the WGD-dgps 75.09% underwent purifying selection, and approximately three-quarters of informative WGD-dgps expressed their two paralogs with significant differences. We detected a wide variety of anthocyanins in the berries of *R. rosaefolius*, and their total concentration remained relatively stable during berry development but increased rapidly during the ripening stage, mainly because of the contributions of pelargonidin-3-*O*-glucoside and pelargonidin-3-*O*-(6″-*O*-malonyl)glucoside. We identified many structural genes that encode enzymes, such as RrDFR, RrF3H, RrANS, and RrBZ1, and play key roles in anthocyanin biosynthesis. The expression of some of these genes significantly increased or decreased with the accumulation of pelargonidin-3-*O*-glucoside and pelargonidin-3-*O*-(6″-*O*-malonyl)glucoside. We also identified some transcription factors and specific methylase-encoding genes that may play a role in regulating anthocyanin biosynthesis by targeting structural genes. In conclusion, our findings provide deeper insights into the genomic evolution and molecular mechanisms underlying anthocyanin biosynthesis in berries of *R. rosaefolius*. This knowledge may significantly contribute to the targeted domestication and breeding of *Rubus* species.

## Introduction

*Rubus* (Rosaceae) contains 12 subgenera with more than 700 species that are scattered around the world except Antarctica [1, 2]. Eastern Asia or western North America is speculated to be the origin center, and southwestern China is hypothesized to be the diversity center of *Rubus* species [1, 3]. Tissues, especially mature berries, of *Rubus* species usually contain a variety of health-promoting compounds (e.g. anthocyanins, phenolic acids, and flavonoids) and nutrient substances (e.g. cellulose, vitamin E, and natural pigments) [4, 5]. They have been used as medicine and fruit since ancient times [6]. Some *Rubus* species (e.g. *Rubus occidentalis* and *Rubus idaeus*) have been domesticated and cultivated, and their planting area and production have increased rapidly in the past two decades [7]. Although most *Rubus* fruits are underutilized despite their high commercial value, cultivating these fruits can better satisfy people’s needs. Many researchers have investigated *Rubus*, and studies have focused mainly on taxonomy [2, 8], phytochemistry, pharmacology [9, 10], and genomics [11, 12]. Information on the genomic evolution and molecular basis of important biological properties of *Rubus* species is limited to a few members.

**Table 1:** Listing of genome assembly indices of four *Rubus* species

Species name	Genome assembly index					No. of references
	Coverage ratio (%)	Number of contigs	N50 of contig	Number of scaffolds	N50 of scaffolds	
*R. rosaefolius*	98.68	138	16.13 Mb	131	30.0 Mb	
*R. occidentalis*	83			2226	353 kb	24
	98.98	235	5.1 Mb			25
	76.38	11 936	49.49 kb			11
*R. chingii*	97.70%			155	8.2 Mb	12
*R. idaeus*	99.42%	2350	0.24 Mb			23

*Rubus rosaefolius* (syn. *Rubus rosifolius* Sm.) is a kind of red raspberry that grows naturally in East Asia, Southeast Asia, South Asia, Oceania, Africa, and Madagascar (*Flora of China*: http://www.iplant.cn/foc). The twigs, leaves, and roots of *R. rosaefolius* have long been used for relieving cough, dispelling wind, hemostasis and dampness [13]. The mature berry of *R. rosaefolius* is rich in bioactive ingredients, such as anthocyanins [14], phenolics [15], triterpenes, and sterols [13, 16]. As a result, it has multiple pharmacological effects, including antioxidant [15], analgesic [17], antimicrobial [18], anticorrosive [19], antiproliferative [13], and anticancer effects [16], diuresis [20], and the mitigation of hypertension [21]. Additionally, the mature berry of *R. rosaefolius* has a unique sour–sweet taste and is brightly colored, which is attractive. Overall, *R. rosaefolius* is an appealing wild fruit with high commercial value and, therefore, has great potential for domestication and cultivation.

In this study we sequenced, assembled, and analyzed the genome and the transcriptomes of young roots, young stems, young leaves, calyxes, petals, and stamens of *R. rosaefolius*. Moreover, a comprehensive analysis of the transcriptome and metabolome of berry tissues at the young, coloring, and mature stages was also conducted for *R. rosaefolius*. The main aims were to (i) assess the evolutionary dynamics of the genome of *R. rosaefolius* and (ii) elucidate the molecular basis of anthocyanin biosynthesis in the berries of *R. rosaefolius.* Our findings might provide deeper insights into the evolution of *Rubus* species and a foundation for their domestication, breeding, and improvement in the future.

## Results

### Draft genome assembly

We first constructed and sequenced a short-read library (350 bp) of *R. rosaefolius*, and harvested 59.76 Gb of data and 97.53% of qualified reads (>Q20) (Supplementary Data Table S1). We performed a *K*-mer analysis using these qualified reads and estimated that the genome size of *R. rosaefolius* is 220.50 Mb, with a repetition ratio of 33.31% and heterozygosity of 1.64% (Supplementary Data Table S2; Supplementary Data Fig. S1). This result indicates that the genome size of *R. rosaefolius* was smaller than that of *R. occidentalis* [22], *Rubus chingii* [12], and *R. idaeus* [23]. Then, a long-read library of *R. rosaefolius* was constructed and sequenced, and 672 954 clean long reads with an average length of 17 004 bp were obtained (Supplementary Data Table S3). These long reads were assembled into a preliminary draft genome of 241.76 Mb, which consisted of 141 contiguous sequences (contigs) (N50 = 15.36 Mb) (Supplementary Data Table S4). Finally, we constructed and sequenced a Hi-C library of *R. rosaefolius* and obtained >165 million read pairs (Supplementary Data Table S5). Using these valid interaction read pairs to correct the preliminary draft genome, we obtained a final draft genome with a total of 221.95 Mb, containing 138 contigs (N50 = 16.13 Mb). These contigs were further clustered into 131 scaffolds (N50 = 30.0 Mb) (Table 1, Supplementary Data Table S6). Seventy scaffolds with a total of 219.02 Mb, accounting for 98.68% of the total assembly sequences and 99.33% of the estimated genome size, were anchored to seven pseudochromosomes. Among them, 20 scaffolds (211.23 Mb) that represent 96.44% of anchored sequences were determined with respect to position and direction (Supplementary Data Table S7; Supplementary Data Fig. S2). We estimated the assembly integrity of this draft genome by blasting the cluster of essential genes (CEG) database and the Benchmarking Universal Single-Copy Orthologs (BUSCO) database; 456 (99.56%) homologs of 458 CEGs and 1590 (98.51%) homologs of 1614 BUSCOs were identified (Supplementary Data Tables S8 and S9). We realigned this draft genome with clean, qualified short- and long-read sequencing data and found that 99.29 and 98.89%, respectively, of the draft genome had 10× coverage (Supplementary Data Tables S10 and S11; Supplementary Data Fig. S3). These results suggested that the assembled draft genome of *R. rosaefolius* had high integrity. By combining the indices of assembled completeness, gapless degree, and N50 of the contigs or scaffolds, the assembled draft genome in this study surpassed that of other raspberry species (Table 1) [11, 12, 23–25].

### Draft genome annotation

**Figure 1:**
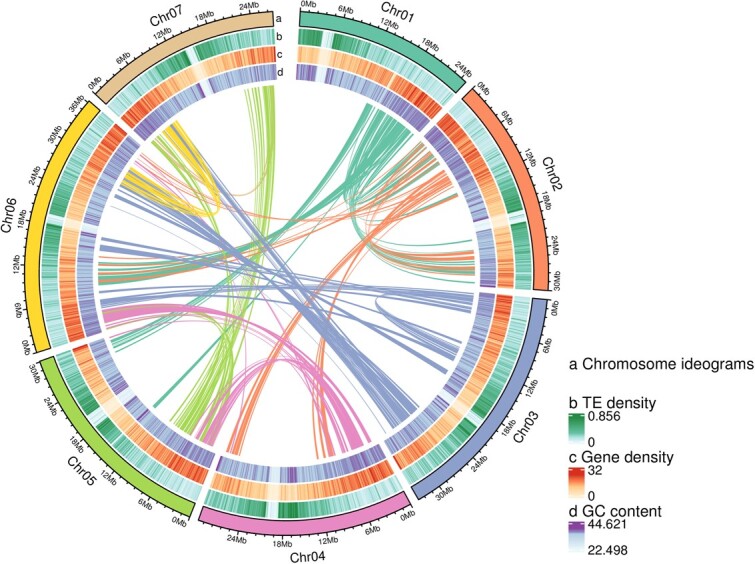
Distribution patterns of transposable elements (TEs), genes and GC content in the chromosomes of *R. rosaefolius.*

From the assembled draft genome of *R. rosaefolius*, we identified 56.39 Mb (25.41%) of interspersed repeats, 68.5% (38.62 Mb) of which were retroelements dominated by the LTR/Copia, LTR/Gypsy, and LINE families, and 31.5% (17.75 Mb) of which were DNA transposons dominated by the PIF-Harbinger, hAT, and Helitron families (Supplementary Data Table S12). We also identified 22.85 Mb of tandem repeat sequences composed of microsatellite, mini-satellite, and satellite units, accounting for 10.29% of the final draft genome sequence (Supplementary Data Table S13). By integrating *ab initio* prediction, homology-based prediction, and RNA-seq prediction, we identified 28 067 protein-coding genes, 3062.13 bp in length, 5.16 exons, and 4.16 introns on average, indicating there were fewer genes in *R. rosaefolius* than in *R. chingii* (28 877 genes), *R. idaeus* (33 865 genes), and *R. occidentalis* (33 248 genes) (Table 1; Supplementary Data Tables S14 and S15; Supplementary Data Fig. S4). Of these predicted protein-coding genes, 1584 (98.14%) were homologs of BUSCOs (1614) (Supplementary Data Table S16), and 26 173 (93.25%) could be annotated for biological or molecular functions (Supplementary Data Table S17; Supplementary Dataset 1). These annotated transposable elements and protein-coding genes were unevenly distributed on the chromosomes of *R. rosaefolius* (Fig. 1)*.* We also identified more than one and a half thousand RNA genes, mainly rRNA and tRNA genes, and some pseudogenes from the *R. rosaefolius* draft genome (Supplementary Data Table S18; Supplementary Dataset 2).

### Phylogenetic relationships and gene family dynamics of *R. rosaefolius* and nine other species

**Figure 2:**
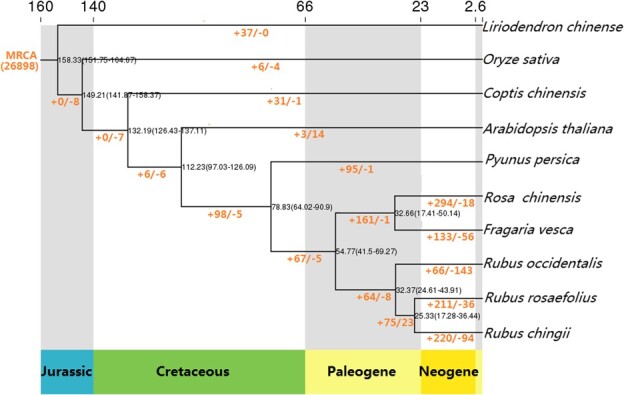
Phylogenetic tree of *Rubus rosaefolius* and nine other species and statistical information on the expansion and contraction of gene families. Time on the evolutionary tree represents the bifurcation time supported by 95% of the highest posterior density (HPD), with the geological time displayed at the bottom of the tree and the absolute time at the top (in million years); ‘+/−’ and digits indicate the numbers of expanded and shrunk gene families, respectively.

To elucidate the evolutionary dynamics of the genome of *R. rosaefolius*, we comprehensively analyzed and compared the gene set data of *R. rosaefolius*, *Coptis chinensis*, *Liriodendron chinense*, *Oryza sativa*, *Arabidopsis thaliana*, *Rosa chinensis*, *Fragaria vesca*, *Prunus persica*, *R. chingii*, and *R. occidentalis.* In total, 242 905 genes, whose encoded proteins contain >100 amino acids, from the above 10 species were clustered into 38 785 orthogroups (families) (Supplementary Data Table S19; Supplementary Dataset 3). Among them, 702 genes that were clustered into 159 families were found to be specific to *R. rosaefolius* (Supplementary Data Table S19; Supplementary Data Fig. S5; Supplementary Dataset 4). These genes were mainly enriched in metabolic pathways such as ‘biosynthesis of amino acids’, ‘other types of *O*-glycan biosynthesis’, ‘glucosinolate biosynthesis’, and ‘valine, leucine, and isoleucine biosynthesis’ (Supplementary Data Fig. S6). These specific families might have imparted some characteristics to *R. rosaefolius* that were different from those of the other nine species. A phylogenetic tree of the above-mentioned species constructed using 901 single-copy gene families (Supplementary Dataset 5) showed that Rosaceae appeared in the Cretaceous period, raspberry appeared in the Paleogene, and *R. rosaefolius* and another red raspberry, *R. chingii*, diverged from the recent common ancestor 17.28–36.44 million years ago (mya), then 211 and 36 gene families of *R. rosaefolius* expanded and contracted, respectively (Fig. 2; Supplementary Dataset 6). The genes in these expanded families were enriched mainly in metabolic pathways, such as ‘DNA polymerase’, ‘photosynthesis’, ‘circadian rhythm’, and ‘galactose metabolism’ (Supplementary Data Fig. S7; Supplementary Dataset 6). Of the 901 single-copy genes used for constructing the phylogenetic tree, 156 (17.31%) underwent positive selection and were enriched in metabolic pathways such as ‘porphyrin and chlorophyll’, ‘biosynthesis of amino acid’, and ‘valine, leucine, and isoleucine biosynthesis’ (Supplementary Data Fig. S8; Supplementary Dataset 5).

### Genome collinearity between *R. rosaefolius* and its five relatives

**Figure 3:**
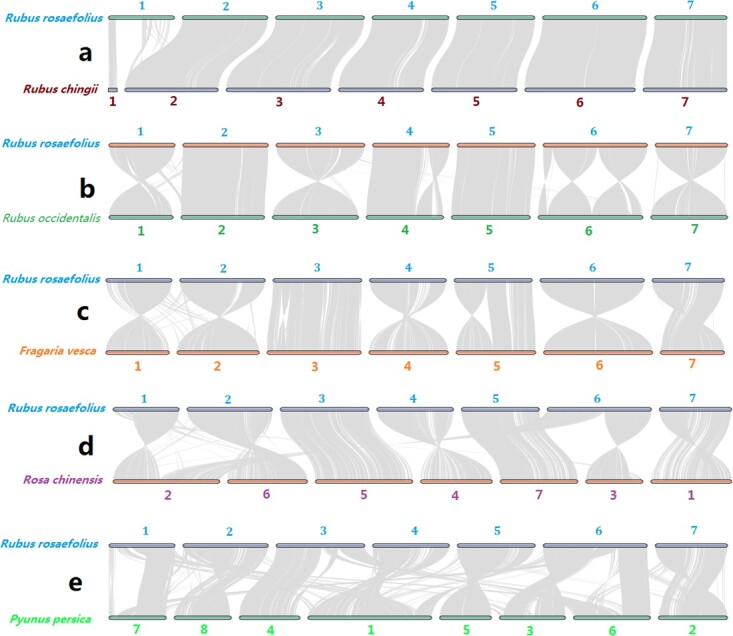
Genome collinearity between *R. rosaefolius* and its relatives. **a***Rubus rosaefolius* versus *R. chingii*; **b***R. rosaefolius* versus *R. occidentalis*; **c***R. rosaefolius* versus *F. vesca*; **d***R. rosaefolius* versus *R. chinensis*; **e***R. rosaefolius* versus *P. persica*.

All plants come from a far or near common ancestor. Therefore, their genomes appear more or less collinear. The pattern of collinearity is strongly influenced by chromosomal variation (e.g. whole or segmental chromosome duplication, loss, inversion, or translocation events) that occurred during an evolutionary process after differentiation from recent common ancestors. In turn, the dynamics of chromosome variation between species can be speculated based on the pattern of genomic collinearity. We found complete and parallel syntenic orthologs at one end of chromosome 1 and on the other six chromosomes of *R. rosaefolius* and *R. chingii*, but macrosyntenic blocks were missing at the other end of chromosome 1 (Fig. 3a). These findings indicated that no large variant of the chromosome occurred between *R. rosaefolius* and *R. chingii*, but a fault was hidden in the assembly or annotation of the draft genome of *R. rosaefolius* or *R. chingii*. We found that the collinearity across all chromosomes between *R. rosaefolius* and *R. occidentalis* was almost intact (Fig. 3b), which indicated that the above-mentioned fault in the assembly or annotation of chromosome 1 probably did not occur in *R. rosaefolius*. When the interference caused by the direction of the scaffolds assembled in the chromosome haplotypes was ignored, nearly complete and parallel collinearity was found for chromosomes 1, 2, 3, 5, and 7, a large inversion probably occurred on chromosome 4, and three or fewer inversions probably occurred on chromosome 6 (Fig. 3b). For *R. rosaefolius* and *F. vesca*, chromosomes 1, 2, 3, 4, and 6 exhibited intact and parallel collinearity, but one end of chromosomes 5 and 7 underwent a large inversion event, the inversion event on chromosome 5 occurred at the same end, while on chromosome 7, it occurred at the opposite end (Fig. 3c). The genomes of *R. chinensis* and *R. rosaefolius*, and particularly the genomes of *P. persica* and *R. rosaefolius*, appeared less collinear than those between *R. rosaefolius* and *R. chingii*, *R. occidentalis*, and *R. chinensis* (Fig. 3a–e)*.* These findings indicated that a greater number of complex chromosomal variations occurred as the kinship distance increased between the species.

### Whole-genome duplication event in *R. rosaefolius*

**Figure 4:**
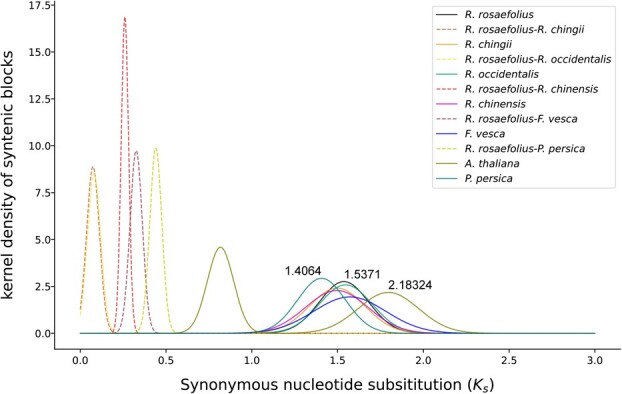
Density distribution of *K*_s_ of paralogs within a species or orthologs between species.

Whole-genome duplication (WGD) plays a key role in adaptation and speciation. The peak density distribution of *K*_s_ (synonymous substitution ratio) of syntenic paralogs within a species usually indicates a WGD event, whereas those of syntenic orthologs between species indicate differentiation events, which are similar to WGD events, from their recent common ancestor [26]. The *K*_s_ value at the top (top^*K*s^) of the corresponding peak is usually positively correlated with the age of the WGD or differentiation event that occurred in the past. We identified one peak (top^*K*s^ = 1.5371) in the *K*_s_ density distribution based on the syntenic paralogs of *R. rosaefolius*–*R. rosaefolius* (Fig. 4). A roughly similar peak also appeared for both *K*_s_ density distributions based on the orthologs of *R. chingii*–*R. chingii*, *R. occidentalis*–*R. occidentalis*, *R. chinensis–R. chinensis*, *F. vesca–F. vesca*, *P. persica–P. persica* and *R. chinensis*–*R. chinensis* with top^*K*s^ = 1.4064–1.5371, which were all larger than the top^*K*s^ of syntenic orthologs of *R. occidentalis* versus the other above-mentioned Rosaceae members (Fig. 4)*.* These results indicated that a WGD event had occurred in their common ancestor, and these findings supported the conclusion that a WGD event was shared among the members of the Rosaceae family, as proposed by Xiang *et al*. [27].

### Evolutionary dynamics of duplicated gene pairs

Duplicated gene pairs (dgps) that arose from WGD events have extensive genetic and evolutionary effects [28]. Hence, their evolutionary dynamics and fate can be utilized. We identified 5090 dgps in the *R. rosaefolius* genome that occurred due to WGD (Supplementary Dataset 7). After removing those dgps whose single-gene expression was zero, 76.90, 73.77, 76.70, 77.69, 73.03, 75.17, 73.44, 74.67, and 79.44% of the WGD-dgps exhibited significant differences in expression between two single genes in nine tissues, namely the young roots, young leaves, young stem, petals, calyxes, stamens, young berries, coloring berries, and mature berries, respectively (Supplementary Data Fig. S9a–i; Supplementary Data Table S20). These findings suggested that the divergent evolution between the paralogs of most WGD-dgps led to significant divergent expression. Of the 5090 WGD-dgps, 53 (1.04%) had a *K*_a_/*K*_s_ value >1 and exhibited strong positive selection, and 298 (5.85%) had a *K*_a_/*K*_s_ value of 0.5–1.0, which suggested that these pairs underwent weak positive selection. However, 3822 (75.09%) pairs had a *K*_a_/*K*_s_ value <0.5, which indicated that they underwent negative selection (Supplementary Data Fig. S9j; Supplementary Dataset 7). These results indicated that purifying selection resulting in the filtering of harmful mutants was the main evolutionary driver of WGD-dgp.

### Anthocyanins in berries and their accumulation dynamics

**Figure 5:**
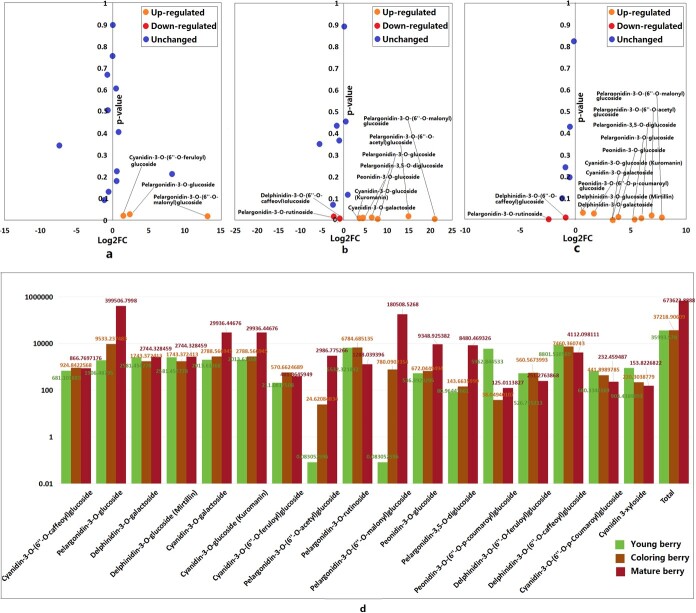
Significant differences in anthocyanins in berries at different stages. **a** Comparing young with coloring stages. **b** Comparing young with mature stages. **c** Comparing coloring with mature stages. **d** Relative concentration in young, coloring, and mature berries.

Previous studies have demonstrated that anthocyanins are abundant in the berries of *R. rosaefolius*, particularly cyanidin and pelargonidin [15, 29]. In this study, we utilized UPLC–MS/MS wide-targeted metabolite profiling to measure the levels of 1330 metabolites, including 121 amino acids and derivatives, 248 phenolic acids, 241 flavonoids, 78 alkaloids, and 151 terpenoids and organic acids (Supplementary Dataset 8). Among the flavonoids, 17 anthocyanins were detected in nine berries at different stages: three at the young (N1) stage, three at the coloring (N2) stage, and three at the mature (N3) stage (Supplementary Dataset 8). During the transition from N1 to N2 (representing the developmental stage), the concentrations of total and most kinds of anthocyanins remained relatively stable, and only pelargonidin-3-*O*-glucoside, cyanidin 3-xyloside, and pelargonidin-3-*O*-(6″-*O*-malonyl)glucoside continued to increase (Fig. 5; Supplementary Dataset 8). From N2 to N3 (representing the developmental stage), the concentrations of five kinds of anthocyanins (e.g. pelargonidin-3-*O*-(6″-*O*-acetyl)glucoside and peonidin-3-*o*-glucoside) remained relatively stable, and two kinds of anthocyanins (pelargonidin-3-*O*-rutinoside and delphinidin-3-*O*-(6″-*O*-caffeoyl)glucoside) slightly decreased; in addition, the total and 10 other kinds of anthocyanins showed increasing trends. Eight (e.g. cyanidin-3-*O*-galactoside and pelargonidin-3-*O*-(6″-*O*-acetyl)glucoside) increased slightly, and two (pelargonidin-3-*O*-glucoside and pelargonidin-3-*O*-(6″-*O*-malonyl)glucoside) increased sharply (Fig. 5; Supplementary Dataset 8). These results clearly indicate that the production of anthocyanins in *R. rosaefolius* berries occurs primarily during the ripening stage, and the main contributors are the reddish pigments pelargonidin-3-*O*-glucoside and pelargonidin-3-*O*-(6″-*O*-malonyl)glucoside.

### Structural genes related to biosynthesis of anthocyanins

**Figure 6:**
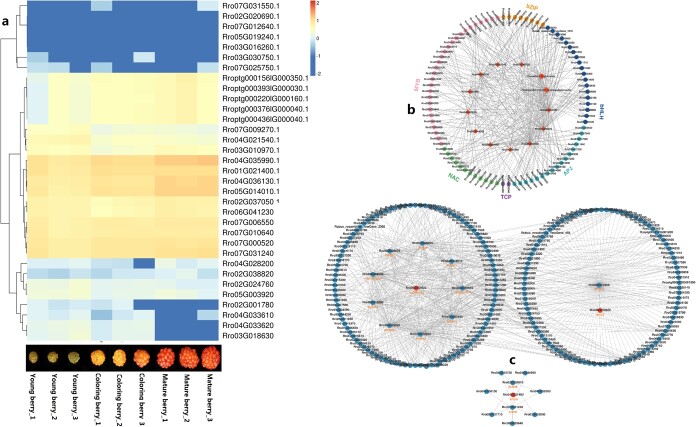
Expression and regulation of structural genes related to anthocyanin biosynthesis. **a** Heat map of gene expression level (log_10_fpkm). **b** Correlation network diagram of differentially accumulated metabolites and differentially expressed genes. **c** Correlation network diagram indicating protein–protein interactions. Solid lines indicate a positive correlation, while dashed lines indicate a negative correlation

A total of 23 997 genes were expressed at different levels in *R. rosaefolius* berries (Supplementary Dataset 9). According to the KEGG annotation, we identified 11 structural genes, including six anthocyanidin 3-*O*-glucosyltransferase (RrBZ1) genes belonging to five families; two anthocyanidin 3-*O*-glucoside 2″-*O*-xylosyltransferase (RrUGT79B1) genes belonging to one family; one anthocyanidin 3-*O*-glucoside 5-*O*-glucosyltransferase (RrUGT75C1) gene and two anthocyanidin 3-*O*-glucoside 6″-O-acyltransferase (Rr3AT) genes belonging to two families, which are involved in the ‘anthocyanin biosynthesis’ pathway. Furthermore, we identified 22 structural genes, including 10 chalcone isomerase (RrCHI) genes, which belong to six families; five chalcone synthases (RrCHS) genes, which belong to four families; four bifunctional dihydroflavonol 4-reductase/flavanone 4-reductase (RrDFR) genes, which belong to three families; one flavonoid 3′-monooxygenase (RrCYP75B1) gene; one naringenin 3-dioxygenase (RrF3H) gene; and one anthocyanidin synthase (RrANS) gene (Supplementary Data Fig. S10; Supplementary Dataset 10), which are involved in ‘flavonoid biosynthesis’ and play crucial upstream roles in anthocyanin biosynthesis [30]. Of these 33 structural genes, *Rro07G000520.1* (RrBZ1), *Rro04G036130.1* (RrBZ1), *Rro01G021400.1* (RrF3H), *Rro07G031240.1* (RrDFR), *Rro04G035990.1* (RrCHS), *Rro04G036130.1* (RrCHS), *Rro07G006550.1* (RrCHI), and *Rro05G014010.1* (RrANS) were expressed at relatively high levels in berry tissues (Fig. 6a; Supplementary Dataset 10). We speculate that these eight genes are the main players involved in the biosynthesis of anthocyanins in the berries of *R. rosaefolius.* A total of 33 structural genes were also expressed in the flower, leaf, and root tissues of *R. rosaefolius*, but none were exclusively expressed in berries (Supplementary Dataset S7). This finding indicates that anthocyanin metabolism occurs in various tissues of *R. rosaefolius* and is not limited to berries alone. The above-mentioned 33 structural genes could be clustered into 25 families, with only one gene family (*OG0002772*) being involved in the expansion/construction of gene families in the genomes of the seven Rosaceae species used for comparison (Supplementary Datasets 6 and 10). This result provided evidence that these structural genes are evolutionarily conserved in the Rosaceae. Gene duplication events were observed in a few families, including those encoding RrBZ1 (*Rro04G033610.1* and *Rroo4G033620.1*) and RrUGT79B1 (*Rro03G016260.1* and *Rro04G021540.1*) (Supplementary Dataset 10). Phylogenetic analysis of these gene families suggested that RrBZ1-dgp arose from a WGD event, while RrUGT79B1-dgp originated from a *Rubus* lineage-specific duplication event (Supplementary Data Fig. S11). However, all these dgps express paralogs at varying levels in berries (Supplementary Data Fig. 6a), indicating differential evolutionary fates for the paralog of these dpgs and contributing to the complexity of anthocyanin biosynthesis and regulation.

### Gene regulation of anthocyanin biosynthesis

To explore the molecular mechanism regulating anthocyanin biosynthesis in the berries of *R. rosaefolius*, we conducted a correlation analysis between the transcriptomes and metabolomes of berries at the young and mature stages. Peonidin-3-*O*-glucoside and pelargonidin-3-*O*-(6″-*O*-malonyl)glucoside, the major anthocyanins found in mature berries as previously stated, were identified as significant differentially accumulated metabolites (DAMs), and 11 of the above-mentioned structural genes were found to be differentially expressed genes (DEGs). Of these DEGs, *Rro07G000520.1* (RrBZ1), *Rro04G035990.1* and *Rro04G036130.1* (RrCHS), *Rro07G006550.1* (RrCHI), *Rro01G021400.1* (RrF3H), *Rro07G031240.1* (RrDFR), and *Rro05G014010.1* (RrANS) were positively correlated with the accumulation of peonidin-3-*O*-glucoside and pelargonidin-3-*O*-(6″-*O*-malonyl)glucoside, while *Rro04G033620.1* and *Rro02G024760.1* (RrBZ1), *Rro07G009270.1* (RrUGT75C1), and *Rro04G021540.1* (RrUGT79B1) were negatively correlated with these two DAMs (Fig. 6b). The expression of several DEGs that encode transcription factors that belong to the MYB, bHLH, AP2, bZIP, NAC, and TCP families was significantly positively or negatively correlated with one or more of the above-mentioned structural genes and two DAMs (Fig. 6b). These findings imply that transcription factors are widely involved in the regulation of anthocyanin biosynthesis in the berries of *R. rosaefolius.* By protein–protein interaction (PPI) analysis, we observed that the *Rro4G033620* protein (RrBZ1) directly interacts with the enzyme trimethylguanosine synthase 1 (RrTGS1); that the *Rro5G003920* protein (RrBZ1) directly interacts with ferredoxin C1 (RrFDX1), CCAAT-binding transcription factor (RrNFYA), glutamate synthase 1 (RrGLU1) and histone-like transcription factor (RrCBF/NF-Y); and that the *Rro1G021400* protein (RrF3H) directly interacts with RrFDR and RrANR (Fig. 6c). These results suggested that anthocyanin biosynthesis in the berries of *R. rosaefolius* was regulated by RNA methylation, the interaction of proteins that were encoded by structural genes, and transcription factors.

## Discussion

The genus *Rubus* contains more than 700 species [1, 2]. This number is considerably greater than the average number of members at the genus level in the plant kingdom (67; http://www.theplantlist.org/statistics/). Therefore, *Rubus* has probably experienced evolutionary radiation. Complex apomixis, polyploidization, and frequent hybridization have been found in *Rubus* species [1, 2, 8]. These events can at least partly explain the biological and genetic mechanisms underlying the relatively frequent speciation events that have occurred in the *Rubus* genus. In this study, a few large chromosome inversions were found between the genomes of *R. rosaefolius* and *F. vesca* according to collinearity analysis, suggesting that large chromosome inversion events occurred in the common ancestor of the *Rubus* lineage or in the common ancestor of the *Fragaria* lineage. Large chromosome inversion events, which are highly likely to have occurred in the ancestor of the *Rubus* lineage, caused unstable inheritance of the genome, which differentiated rapidly and was therefore likely to also promote the evolutionary radiation of *Rubus* species as well as apomixis, polyploidization, and hybridization. WGD and other genetic mechanisms, such as transposon activity and asymmetric replication of chromosomes, produce a large number of dgps, which provide key resources for producing new genes, allowing the organism to acquire genetic novelty [28]. We identified 5090 WGD-dgps from the draft genome of *R. rosaefolius*, which was smaller than the number produced by theoretical WGD events. Hence, most dgps of *R. rosaefolius* that were produced by WGD events have silenced one or two genes during the evolutionary process. A neutrality test revealed that about three-quarters of the WGD-dgps underwent purifying selection. Therefore, solely based on the above results, the fate of duplicated gene pairs that occurred due to WGD in *R. rosaefolius* matched that of *Homo sapiens*, *Caenorhabditis elegans*, *Drosophila melanogaster*, *A. thaliana*, *Schizosaccharomyces pombe*, and *Saccharomyces cerevisiae*, as determined by Lynch and Conery [31]*.* Of the informative WGD-dgps, 73.03–79.44% exhibited significant divergence between two members in the roots, leaf, stem, petals, calyxes, stamens, young berry, coloring berry, and mature berry tissues. These results were basically consistent with those for sweet cherry, in which 75% of WGD-dgps were found to diverge in expression at different bud development stages [32]. In summary, there is a strong consistency in the evolutionary dynamics of WGD-dgps across different species.

Anthocyanins are crucial flavonoid phytochemicals found in various plant tissues, contributing to the coloration of plant tissues and playing essential roles in physiological functions [33]. These compounds hold significant nutritional and health value for humans [34]. Most raspberry species are known for their anthocyanin content [14, 35, 36]. According to the report of Bowen-Forbes *et al*. [29], cyanidin and pelargonidin were the primary anthocyanins in *R. rosaefolius* berries. In our current research, four types of anthocyanins (pelargonidins, peonidin, cyanidins, and delphinidins) were detected in *R. rosaefolius* berries. The total anthocyanin content is stable from the young to the coloring stage, during which, together with two reddish pigments, pelargonidin-3-*O*-glucoside, and pelargonidin-3-*O*-(6″-*O*-malonyl)glucoside, increase sharply in ripe berries. These findings suggested that the biosynthesis of anthocyanins occurs mainly in the mature stage and that the red coloration of the berries results from the accumulation of reddish anthocyanins, which is different from that of another red raspberry, *R. chingii*, whose anthocyanin content decreased during berry maturation [36]. Moreover, many organic acids, amino acids and derivatives, vitamins, alkaloids, terpenoids, and flavonoids were found in the mature berries of *R. rosaefolius.* Therefore, *R. rosaefolius* is a kind of wild fruit with wide nutritional and health benefits and deserves to be domesticated into a new fruit crop.

Rosaceae contains many important fruit crops, such as apples, cherries, peaches, strawberries, and raspberries. These fruits are usually rich in color, mainly due to the different types and contents of anthocyanins they contain. The molecular mechanisms involved in the biosynthesis and regulation of anthocyanins in Rosaceae fruits, including those of a few *Rubus* species, has been extensively explored. These studies included the identification of the structural genes (e.g. F3H, DFR, ANS, and 3GT) and regulatory genes (e.g. MYB and bHLH) required for anthocyanin production; the correlation between the accumulation of anthocyanins and the expression of regulatory and structural genes; and the influence of the light and temperature environment and plant hormones on anthocyanin biosynthesis [36–41]. In addition, miRNAs, long non-coding RNAs, and epigenetic mechanisms also affect anthocyanin biosynthesis in Rosaceae fruits by targeting regulatory or structural genes [42–45]. Here we identified all key structural genes related to anthocyanin production in *R. rosaefolius* and found their evolutionary conservatism in the Rosaceae family. This finding suggests that the molecular mechanisms underlying anthocyanin synthesis and regulation in Rosaceae plants may be largely consistent. We observed that the expression of several structural genes, including the RrF3H and RrDFR genes, and the accumulation of anthocyanins both increased significantly in mature berries of *R. rosaefolius*. This positive correlation between the DFR and F3H genes and anthocyanins has been reported in *Rubus coreanus* Miquel berries [37]. We found some genes encoding transcription factors, including MYB and bHLH, whose expression significantly increased or decreased, respectively, with the accumulation of anthocyanin biosynthesis, implying that these transcription factors play a role in the regulation of anthocyanin biosynthesis in the berries of *R. rosaefolius*, similar to their roles in *R. idaeus* [38], *Rubus genevieri* [39], *R. occidentalis* [41], apple [43], and peach [46]. We observed that several structural genes encoding the same protein play different roles in anthocyanin biosynthesis. For example, six RrBZ1 genes were identified in the genome of *R. rosaefolius*, but they were expressed at significantly different levels in berries and even exhibited opposite correlations with anthocyanin biosynthesis. Moreover, we have evidence that the mechanism of RNA methylation and the interaction of proteins that are encoded by structural genes were involved in anthocyanin biosynthesis in the berries of *R. rosaefolius.* The above findings indicate that the biosynthesis of anthocyanins in the berries of *R. rosaefolius* is co-regulated by multiple molecular mechanisms. In summary, we provided a multidimensional perspective on the genetic foundation and molecular mechanisms underlying anthocyanin biosynthesis and regulation in *R. rosaefolius*, particularly in relation to color development during berry ripening. This knowledge helps to characterize anthocyanin synthesis and regulation in Rosaceae fruits and holds great potential for targeted domestication and breeding efforts in *Rubus* species, even Rosaceae species, leveraging biotechnological measures such as genome editing technology.

## Conclusions

In this investigation, a high-quality chromosome-level draft genome was unveiled, containing 35.7% repetitive sequences and 28067 protein-coding genes. A Rosaceae lineage-specific WGD event was traced, resulting in 5090 presently detectable dgps, with most undergoing purifying selection. The accumation of anthocyanins in mature berries of R. rosaefolius is predominantly attributed by pelargonidin-3-O-glucoside and pelargonidin-3-O-(6″-O-malonyl)glucoside. Numerous structural genes responsible for anthocyanin biosynthesis were identified, with their expression possibly regulated by transcription factors and genetic mechanisms such as RNA methylation and protein interactions encoded by structural genes. Overall, these findings could significantly aid in the targeted domestication and breeding of *R. rosaefolius* and other *Rubus* species.

## Materials and methods

### Plant materials

An individual *R. rosaefolius* plant planted in a raspberry germplasm nursery located in Guizhou Botanical Garden was used for genome, transcriptome, and metabolome experiments. The young leaves were subjected to DNA extraction for genome analysis. Young leaves, young stems, young roots, calyx, petals, stamens, and three young berries with 5, 8, and 11 developmental days, respectively; three coloring berries with 14, 17, and 19 developmental days, respectively; and three mature berries with 21, 23 and 25 developmental days, respectively, were subjected to RNA extraction for transcriptome analysis. The same berries were also used to determine the metabolites for metabolome analysis.

### DNA and total RNA extraction, library construction, and sequencing

DNA was extracted using a plant genomic DNA extraction kit (Thermo Fisher Scientific, Inc., Rockford, IL, USA) following the manufacturer’s protocol. Total RNA was extracted using the RNeasy Plant Mini Kit (Qiagen, Hilden, Germany) following the manufacturer’s guidelines. The quality and content of the extracted DNA and total RNA were checked using a Qubit 2.0 fluorometer (Thermo Fisher Scientific, Waltham, MA, USA) and an Agilent 2100 bioanalyzer (Agilent Technologies, Santa Clara, CA, USA). The genomic short-read library, genomic long-read library, short-read transcriptome library, and full-length transcriptome library were constructed following the standard protocol provided by the sequencer manufacturer and have been ‘described earlier [47, 48]. The Hi-C library of *R. rosaefolius* was constructed following the protocols proposed by Rao *et al*. [49]. The qualified genomic short-read library, Hi-C library and short-read transcriptome library were sequenced using a HiSeq 4000 sequencer (Illumina Inc., San Diego, CA, USA) with the PE150 model. The qualified genomic long-read library and full-length transcriptome library were sequenced using a Pacific Biosciences RSII system via single-molecule real-time (SMRT) technology (PacBio, Menlo Park, CA).

### *K*-mer analysis using genomic short-read data

Raw genomic short-read data with a Phred score of <20 were considered to be low-quality reads and were filtered. The qualified short reads were used for further evaluation of contamination by BLAST NCBI-BLAST+ 2.9.0 (https://ftp.ncbi.nlm.nih.gov/blast/executables/blast+/LATEST/) against the NCBI-NT database (https://www.ncbi.nlm.nih.gov/) with the parameters -num_descriptions 100 -num_alignments 100 -evalue 1e-05, and by SOAP alignment against the complete chloroplast genome of *Rubus irritans* Focke (MN652919; 1 155 286 bp). The qualified clean short reads were subsequently used for estimating the genome size, heterozygosity, ploidy, and GC content of *R. rosaefolius* via *K*-mer analysis, which was performed using the Toolkit (KAT) program [50].

### Preliminary assembly and evaluation of the contig-level draft genome

The clean higher-quality genomic long reads were preliminarily assembled into a contig-level draft genome using Hifiasm software [51]. Next, purge_dups software [52] was used to remove possible redundant sequences and obtain a refined contig-leveled draft of the genome. To evaluate assembly integrity, the contig-leveled draft genome was blasted against CEGMA (Core Eukaryotic Genes Mapping Approach) v2.5 (https://github.com/KorfLab/CEGMA_v2) and OrthoDB 10 (http://cegg.unige.ch/orthodb) built into BUSCO v4.0 (https://busco.ezlab.org/) by combining the software tblastn (https://blast.ncbi.nlm.nih.gov/Blast.cgi?PROGRAM=tblastn&PAGE_TYPE=BlastSearch&LINK_LOC=blasthome), genewise (v2.4.1) (https://www.ebi.ac.uk/Tools/psa/genewise/), and geneid (https://github.com/guigolab/geneid). To further evaluate the integrity of the assembly and the uniformity of sequencing coverage, the short-read data were realigned into the draft genome using BWA software [53]. Additionally, the long-read data were also realigned into the draft genome using Minimap2 software [54].

### Hi-C assembly

The raw Hi-C sequencing data were cleaned by removing the adapter and primer sequences and discarding the low-quality reads (<Q20). The remaining clean Hi-C data were further evaluated to identify valid interaction pairs and invalid interaction pairs using HiC-Pro v2.10.0 (https://github.com/nservant/HiC-Pro). The valid interaction pairs were subsequently aligned into the contig-level draft genome using the software BWA v0.7.10-r789 (https://sourceforge.net/projects/bio-bwa/). Only the unique paired alignments were used to perform the Hi-C assembly using LACHESIS software [55]. The genome was assembled by Hi-C into chromosomes and cut into bins of 300 000 bp in equal length; the number of Hi-C read pairs between any two bins was considered the signal intensity of the interaction between the bins. Based on the above results, a heat map of the Hi-C assembly was constructed using R software (https://www.r-project.org/).

### Annotation of repetitive sequences

First, we used RepeatModeler2 (v2.0.1) (https://www.repeatmasker.org/RepeatModeler/) to perform *ab initio* prediction using the software packages RECON (v1.0.8) [56] and RepeatScout (v1.0.6) (https://github.com/mmcco/RepeatScout). Then, we used RepeatClassifier and the Dfam (v3.7) database (https://dfam.org/home) to classify the prediction results. Second, we performed *ab initio* prediction of LTRs using the software LTR_Retriever (v2.9.0) [57] based on the results of LTR share v1.5.10 (https://www.zbh.uni-hamburg.de/en/forschung/gi/software/ltrharvest.html) and the LTR_FINDER (v1.07) [58]. The above *ab initio* prediction results were subsequently combined with the known database to obtain the specific repetitive sequence database of this species after removing redundancies. Finally, we used the program RepeatMasker (v4.1.2) [59] to identify the transposon sequences in this genome based on the constructed repetitive sequence database. Tandem repeat sequences were mainly predicted using the microsatellite identification tool (MISA v2.1) [60].

### Prediction of protein-coding genes

Three methods were used to predict protein-coding genes. Augustus (v3.1.0) [61] and SNAP (2006-07-28) [62] were used for *ab initio* prediction. GeMoMa (v1.7) [63] was used for prediction based on homologous species. Second-generation transcriptome prediction relies mainly on transcripts assembled in two ways. One way involves the application of HISAT (v2.1.0) [64] and StringTie (v2.1.4) [65], and gene prediction is performed by GeneMarkS-T (v5.1) [66]. The other way involves obtaining transcripts through the Trinity (v2.11) [67] assembly, followed by the use of PASA (v2.4.1) (https://github.com/PASApipeline/PASApipeline/releases) for gene prediction. The third-generation transcriptome was compared with that of gmap (http://research-pub.gene.com/gmap/) to process the splicing sites, after which the results were predicted with PASA (v2.4.1). Finally, EVM (v1.1.1) [68] was used to integrate the prediction results obtained from the above three methods, and PASA (v2.4.1) was used for modification. The Embryophyta database in BUSCO (v5.2.2) was used to evaluate the integrity of the gene predictions.

### RNA-seq and pseudogene prediction

The tRNA genes were identified via tRNAscan SE (v1.3.1) (https://github.com/UCSC-LoweLab/tRNAscan-SE). The prediction of rRNA was performed mainly using barrnap (v0.9) (https://github.com/tseemann/barrnap); miRNAs, snoRNAs, and snRNAs were predicted based on the Rfam (v14.5) [69] database and using Infernal (v1.1) [70]. By comparing the data using GenBlastA (v1.0.4) [71], we searched for homologous gene sequences (possible genes) in the genome with the real locus shielded, and then we used GeneWise (v2.4.1) to search for immature stop codons and frameshift mutations in the gene sequences to identify pseudogenes.

### Annotation of predicted protein-coding genes

The predicted protein-coding genes were functionally annotated by blasting with a *P* value (<1E5) against the databases of NR (https://www.ncbi.nlm.nih.gov/refseq/about/nonredundantproteins/), eggNOG (http://eggnog5.embl.de/#/app/home), GO (http://geneontology.org/), KEGG (https://www.genome.jp/kegg/), InterPro (https://www.ebi.ac.uk/interpro/), TrEMBL (http://www.bioinfo.pte.hu/more/TrEMBL.htm), KOG (http://genome.jgi-psf.org/help/kogbrowser.jsf), Swiss-Prot (https://www.expasy.org/resources/uniprotkb-swiss-pro), and Pfam (http://pfam-legacy.xfam.org/).

Nine other species were used for comparison of the genomic analysis with *R. rosaefolius.* To provide deeper insights into the evolutionary dynamics of the genome of *R. rosaefolius*, we conducted a comparative analysis of the genomes of *R. rosaefolius* and *A. thaliana* (https://www.arabidopsis.org/download/index-auto.jsp?dir=%2Fdownload_files%2FGenes%2FTAIR10_genome_release%2FTAIR10_chromosome_files), *F. vesca* (genome v4.0: https://www.rosaceae.org/tools/jbrowse), *R. occidentalis* (v3.0: https://www.rosaceae.org/tools/jbrowse), *Coptis chinensis* (https://ftp.ncbi.nlm.nih.gov/genomes/all/GCA/015/680/905/GCA_015680905.1_ASM1568090v1/GCA_015680905.1_ASM1568090v1_genomic.gff.gz), *Liriodendron chinensis* (https://treegenesdb.org/FTP/Genomes/Lich/v1.0/annotation/), *P. persica* (v2.0:http://genome.jgi.doe.gov/pages/dynamicOrganismDownload.jsf?organism=Ppersica), *R. chingii* (v1.0: //www.rosaceae.org/rosaceae_downloads/Rubus_chingii/rubus_chingii_v1/assembly/Rubus_chingii_Hu_fina.fa.gz), *R. chinensis* (v2.0: https://www.rosaceae.org/tools/jbrowse), and *O. sativa* (Build_4.0: https://www.ncbi.nlm.nih.gov/genome/10).

### Cluster analysis of gene families

Orthofinder v2.4 software (https://github.com/davidemms/OrthoFinder/releases) was used to classify the protein sequences of the above 10 species into families with a diamond alignment and an e-value of 0.001. The PANTHER V15 database (http://www.pantherdb.org/) was used to annotate the obtained gene families.

### Construction of evolutionary tree

Using 901 single-copy gene sequences, an evolutionary tree was constructed using the maximum likelihood method (bootstrap = 1000) via IQ-TREE v1.6.11 software (http://www.iqtree.org/release/v1.6.11). We set the outgroup as ‘L Chinese’ to obtain a rooted tree and then used the software package MCMCTree in PAML v4.9i (https://hpcdocs.kennesaw.edu/modules/Software/PAML/) to calculate the divergence time by referring to the fossil time of *O. sativa* versus *A. thaliana* (143.0–174.8 mya), *R. chingii* versus *R. occidentalis* (28.3–68.8 mya), *F. vesca* versus *C. chinensis* (126.0–136.9 mya), and *L. chinense* versus *A. thaliana* (151.6–164.6 mya) (http://www.timetree.org/). The final evolutionary tree with differentiation time was graphically displayed using MCMCTreeR v1.1 (https://www.rdocumentation.org/packages/MCMCtreeR/versions/1.1).

### Expansion/contraction, selection effect and phylogenetic analysis of the gene family

CAFE v4.2 software (https://github.com/hahnlab/CAFE) was used to estimate the number of gene family members of the ancestors of each branch based on the birth mortality model generated from the evolutionary tree with bifurcation time and the results of gene family clustering. We defined significant expansion or contraction according to the standard that the family-wide *P* values and viterbi *P* values were both <0.05. We used the program package PAML v4.9i for positive selection analysis. For some gene families, we constructed a phylogenetic tree using MEGA software (v4.0) (https://www.megasoftware.net/) with the maximum likelihood method (bootstrap = 1000).

### Collinearity analysis

To perform collinearity analysis, we first used Diamond v2.1.8 (https://github.com/bbuchfink/diamond/releases) to compare the gene sequences of the two species and determine similar gene pairs (e < 1E−5, C score > 0.5). Then, based on the gff3 files of all the compared species, syntenic gene pairs adjacent to each other were identified via the use of the software MCScanX [72]. The genes in all collinear blocks were obtained. The figures show the collinearity of the linear patterns of various species, as determined by JCVI v0.9.13 (https://github.com/tanghaibao/jcvi/wiki).

### Identification and analysis of whole-gene duplication and derivative duplication gene pairs

*K*_s_ analysis for detecting whole-genome duplication events was performed using WGDI software [73]. The frequency of *K*_s_ was plotted using R software. The dgps that arose from WGD events were identified using the software DupGen_finder (https://github.com/qiao-xin/DupGen_finder). The *K*_a_ and *K*_s_ values of WDG-dgp were calculated using the simple *K*_a_/*K*_s_ calculator function of TBtools v1.108 software (https://github.com/CJ-Chen/TBtools/releases). The point graph with *K*_s_ as the horizontal axis and *K*_a_ as the vertical axis and the partition of each data point with slopes of *k* = 1, *k* = 0.5, and *k* = 0.05 was made by the software ggplot2 (https://github.com/tidyverse/ggplot2). The dgps with abnormal extremes and without *K*_a_ or *K*_s_ values were removed.

### GO and KEGG enrichment analysis

All GO enrichment analyses were performed using the GOseq R package based on the Wallenius non-central hypergeometric distribution [74]. The statistical enrichment of the objective gene set in the KEGG pathways was performed using the software KOBAS 2.0 [75].

### Processing of full-length RNA data

The Iso-Seq3 pipeline (https://github.com/PacificBiosciences/IsoSeq) was used to analyze the raw subreads to generate a circular consensus sequence (CCS). The software package CCS v6.2.0 (https://github.com/PacificBiosciences/ccs/releases) was used to polish the CCS subreads. The full-length transcripts were determined when the sequences had poly(A) tails and when the 5′ and 3′ cDNA primers were used. The primers and poly(A) tails of sequencing data were removed using the software packages Lima v2.1.0 (https://github.com/lima-vm/lima/releases) and Iso-Seq3 (https://github.com/PacificBiosciences/IsoSeq), respectively. The high-quality full-length consensus sequences were obtained by using the clustering algorithm Intelligent Clustering Engine, and redundant sequences were removed by mapping to the reference genome with the parameters −ax splice -uf —secondary = no -C5. The mapped reads were further collapsed using the software cDNA_Cupcake v29.0.0 (https://github.com/Magdoll/cDNA_Cupcake) with min-identity = 90% and min-coverage = 85%. After the above processing series was conducted, the transcripts of the full-length transcriptomes were obtained.

### Processing of RNA short reads

The adapter was first filtered from the raw RNA short-reads, and then the poly(A) tails and low-quality reads (Q < 20) were also removed. The remaining high-quality clean reads were used to calculate the content of Q20 and Q30 and the GC content. The clean reads were subsequently mapped to the reference genome and reference full-length transcript using the software HISAT (v2.1.0). Only the reads with a perfect match or one mismatch were further used to reconstruct the transcripts via the software StringTie. The expressed genes were identified by the mapping results. If the reads of an expressed gene were mapped to an existing annotated gene sequence, the gene was considered to be an existing annotated gene and was coded consistently. If the reads of an expressed gene were not mapped to an existing annotated gene sequence but to the full-length transcript (long reads; subreads), the expressed gene was considered to be a novel gene and was recorded as a new identification.

### Quantitative and differential analysis of gene expression

The gene expression levels were estimated by the measure of fragments per kilobase of transcript per million fragments mapped (FPKM). The read counts of each sequenced library were first adjusted through one scaling normalization factor using the package edgeR 4.0.16 (https://bioconductor.org/packages/release/bioc/html/edgeR.html). The differential expression of paralogs of dgps in the roots, stems, leaves, flowers, young berry, coloring berry and mature berry was calculated via EBSeq.v2 software (https://github.com/lengning/EBSeq), and the differential expression of genes between young, coloring and mature berries was estimated by using DESeq2 (https://bioconductor.org/packages/release/bioc/html/DESeq2.html). We determined significant differential expression based on false discovery rate (FDR) < 0.05 and |log_2_(foldchange)| ≥2. We analyzed the PPI network using NetworkAnalyst software (https://www.networkanalyst.ca/) in combination with the PPI database STRING v10 (http://version10.string-db.org/).

### Metabolite extraction, determination, and qualitative and quantitative analysis

The metabolite extraction and determination protocols followed were described by Ying *et al*. [76] and Wang [47], respectively. Metabolite qualitative analysis was conducted using the self-built database GB-PLANT by Biomarker Technologies Co., Ltd (http://www.biomarker.com.cn/biocloud). Isotope signals, duplicate signals containing ions such as Na^+^, K^+^, NH4^+^, and larger fragment ions, were filtered during the analysis. The quantitative analysis of metabolites was performed using triple quadrupole mass spectrometry in multiple reaction monitoring (MRM) mode. For the obtained mass spectrometry data of metabolites in each sample, peak area integration was conducted for all mass spectrometry peaks of metabolites, and the integration of mass spectrometry peaks of the same metabolite in different samples was further corrected [77]. Classification and pathway information of identified metabolites were queried via the KEGG database. The differences in the multiples and significance of each metabolite between the young berry, coloring berry, and mature berry groups, as well as the correlation analysis between the DAMs and DEGs obtained through comparative analysis of the three developmental stages of berry, were calculated using R language packages. The DEG pairs, DAM pairs, and DEG–DAM pairs with Pearson’s correlation coefficient (PCC) ≥ 0.8 and *P* ≤ 0.05 were regarded as strongly correlated. The corresponding visual network diagram linking DEGs and DAMs was drawn using Cytoscape software v3.7.0 [78].

## Acknowledgements

This work was funded by the science and technology plan project of Guizhou Province [(2019) 4318] and [(2021) 239], the science and technology plan project of Guiyang City [2021]3-22, and first-class discipline of Kaili University (Horticulture) [grant number 202102]. We are very grateful to the experimenters and analysts at Biomarker Inc. (www.biomarker.com.cn) who took part in this study.

## Author contributions

Y.S.W. and Q.Y.Z. designed the experiments, J.Y.G. and Q.Y.Z. collected samples and took part in conducting experiments, Y.S.W. and Q.Y.Z. took part in analyzing the data, and Y.S.W. wrote the paper.

## Data availability statement

The draft genome sequence was deposited in the China National Center for Bioinformation Database (https://bigd.big.ac.cn/gsub/, accession number CRA011290). The clean raw data of the long-read and short-read transcriptomes of young stems, young roots, young leaves, calyxes, petals, stamens, young berries, coloring berries, and mature berries were also deposited in the China National Center for Bioinformation Database (https://bigd.big.ac.cn/gsub/, accession numbers CRA011285 and CRA012463).

## Conflict of interest

The authors declare no conflicts of interest.

## Supplementary data

Supplementary data are available at *Horticulture Research* online.

## Supplementary Material

Web_Material_uhae064
