# Supplementary material for: Chromosome-scale genome, together with transcriptome and metabolome, provides insights into the evolution and anthocyanin biosynthesis of Rubus rosaefolius Sm. (Rosaceae)
Source: Hortic Res. 2024 Mar 2;11(4):uhae064. doi: 10.1093/hr/uhae064 (PMC11060340; doi:10.1093/hr/uhae064)
Supplement: Web_Material_uhae064 [file web_material_uhae064.zip › Supplemenary information.docx]

**Supplementary Information**


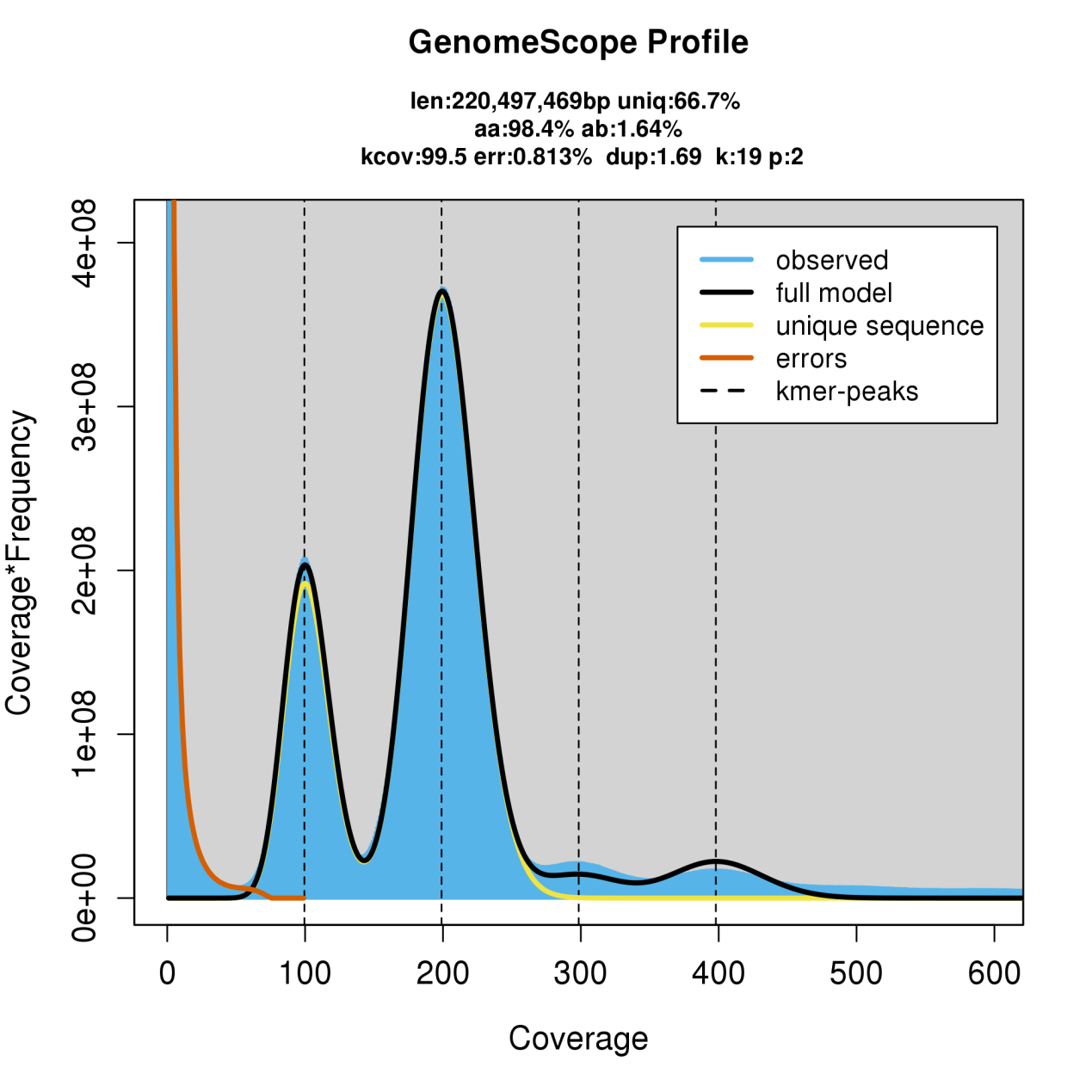


Fig. S1. K-mer distribution and statistics for the genome of *R. rosaefolius*


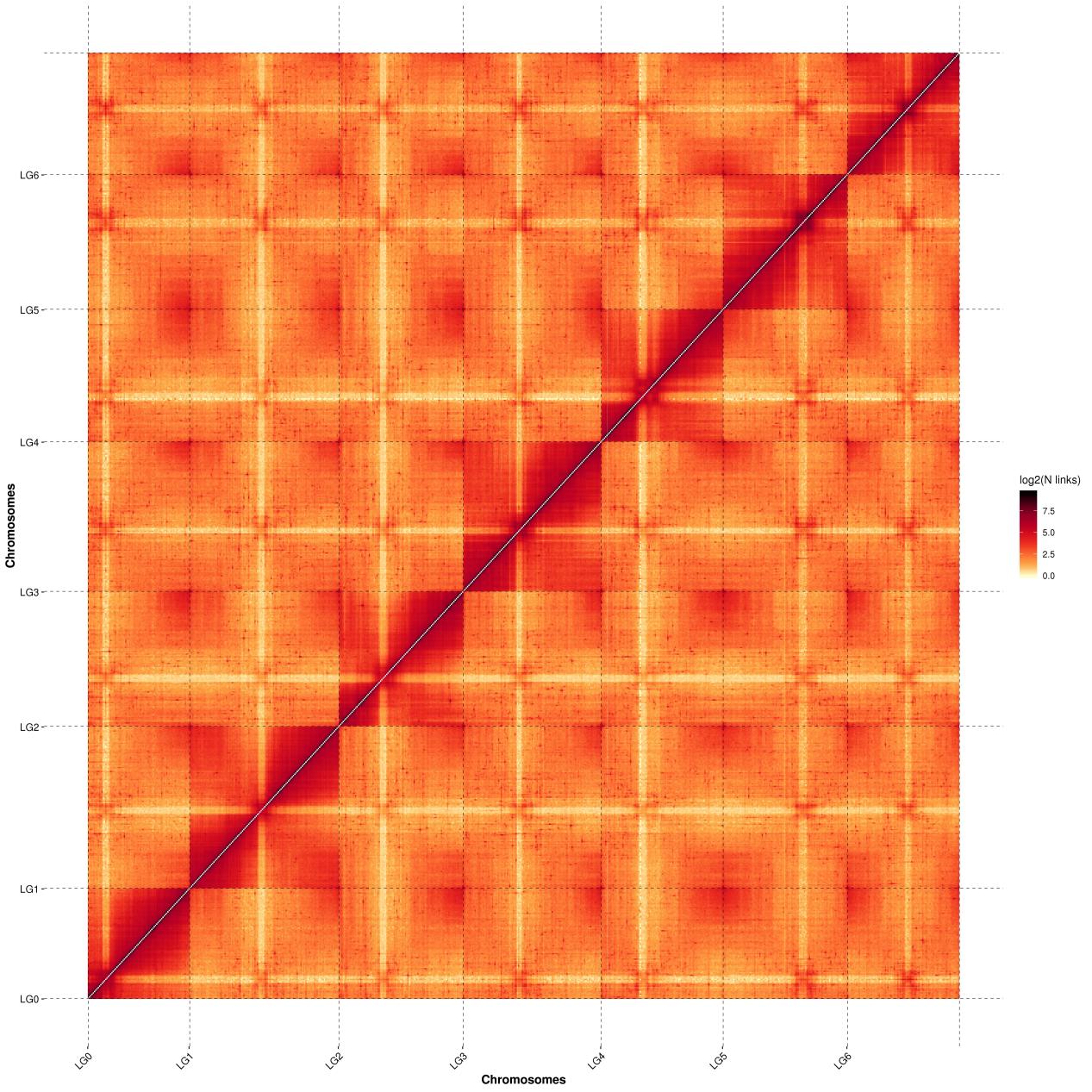


Fig. S2. Heatmap of the Hi-C assembly genome of *R. rosaefolius*


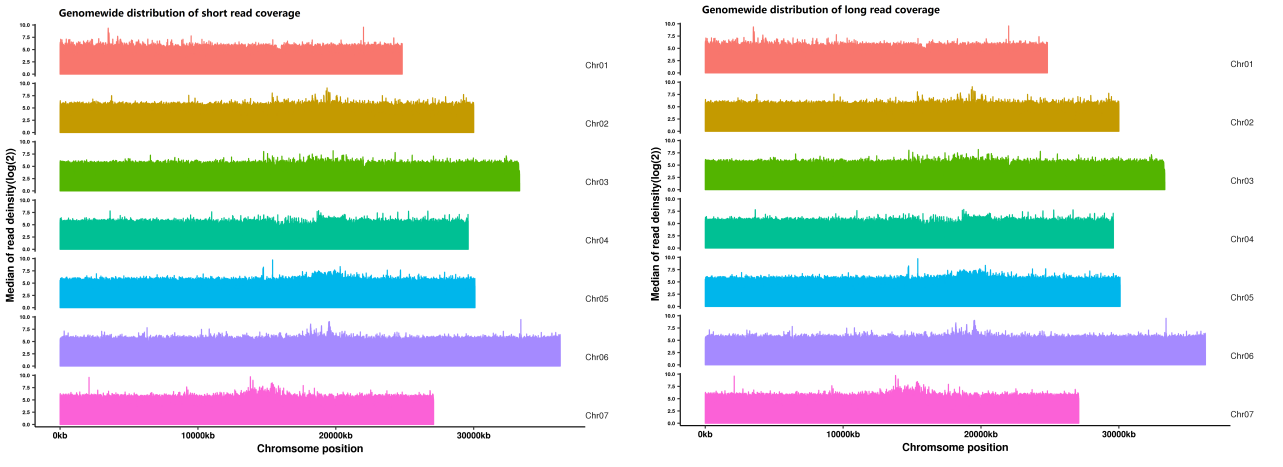


Fig. S3 Genome-wide distribution of read coverage


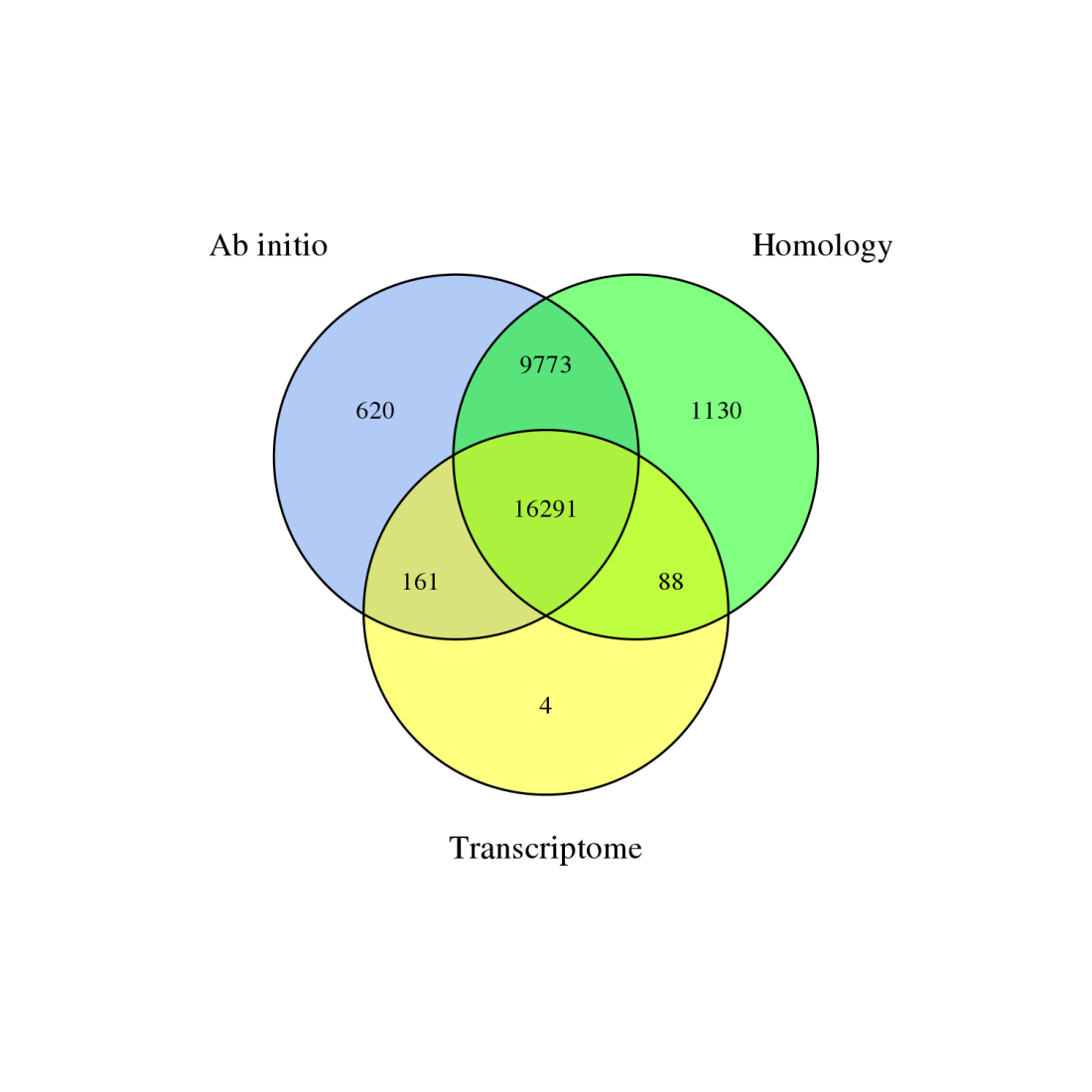


Fig. S4. Venn diagram of the predicted protein-coding genes of *R. rosaefolius* based on three measures


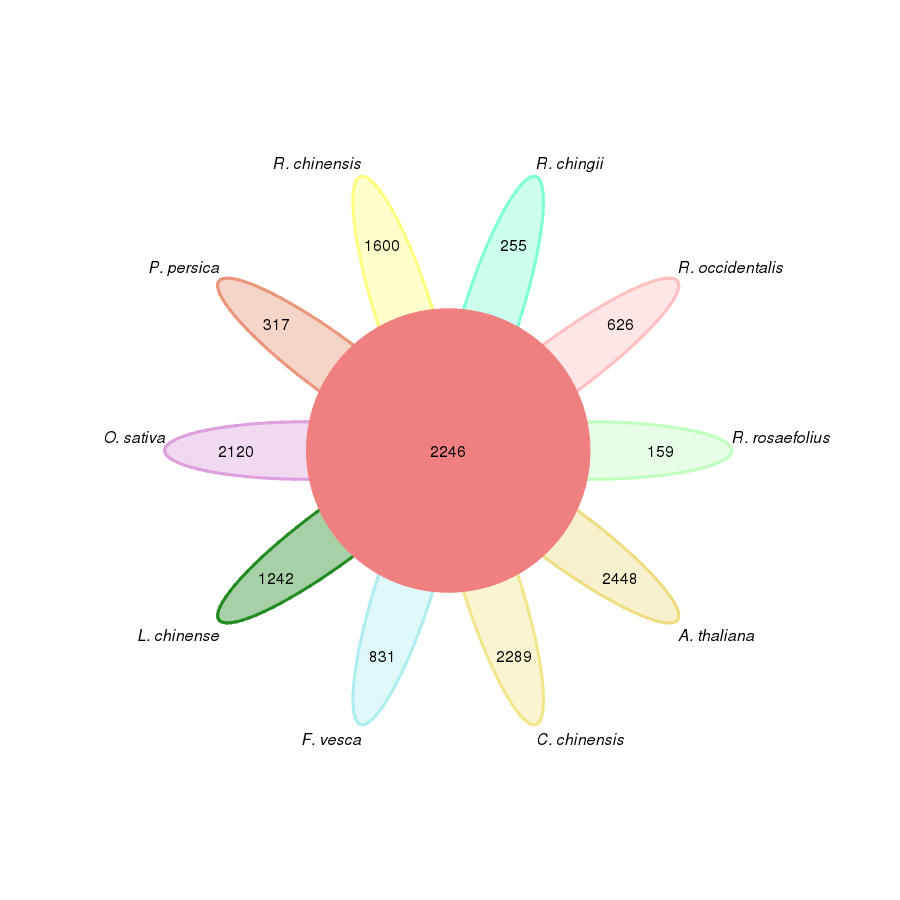


Fig. S5. Statistical information on the specific genes and shared genes of *R. rosaefolius*


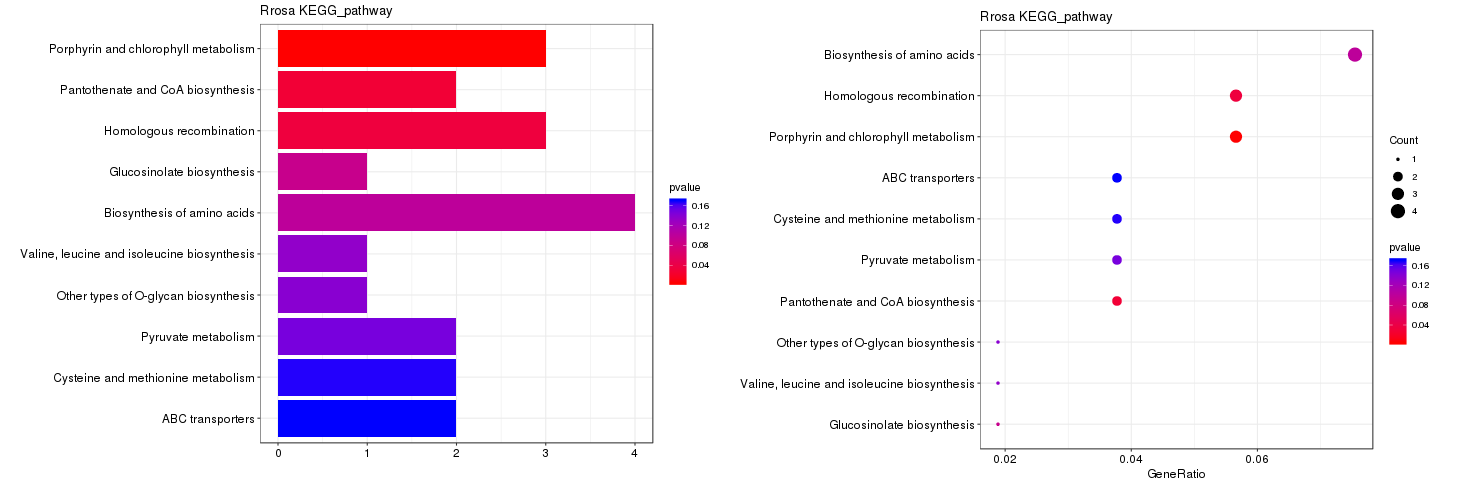


Fig. S6. KEGG enrichment of the specific genes of *R. rosaefolius*


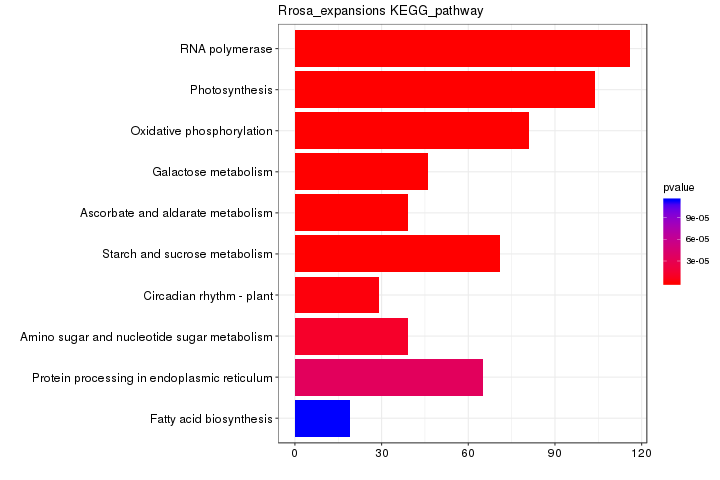


Fig. S7. KEGG enrichment of the genes in the expanded gene families of *R. rosaefolius*


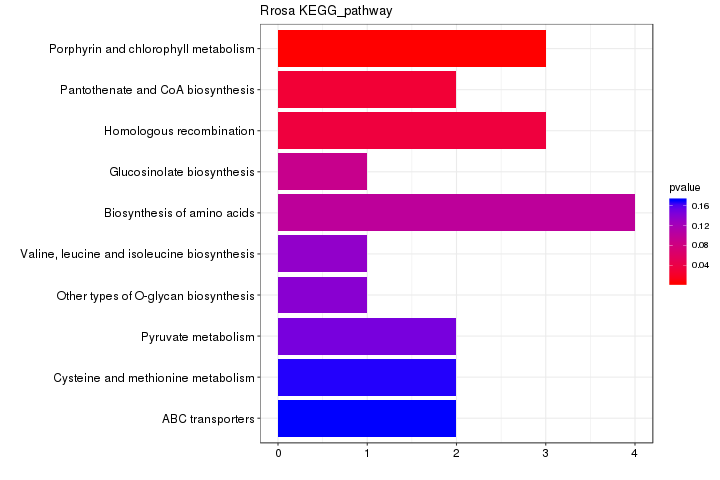


Fig. S8. KEGG enrichment of the single-copy genes of *R. rosaefolius* that underwent positive selection


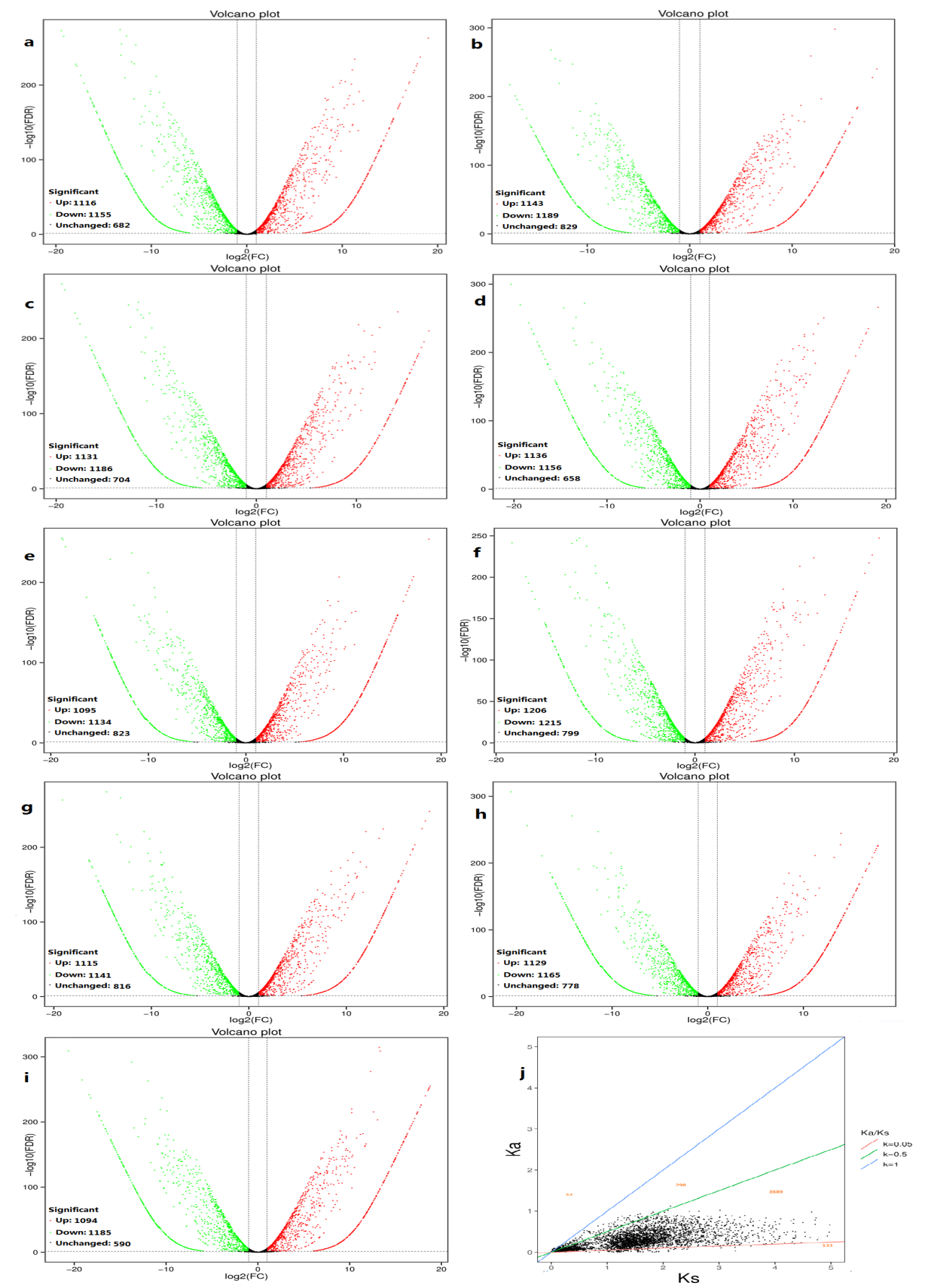


Fig. S9. Statistics of the differences in expression profiles between paired genes of LsDG-dgps in nine tissues and their selection effect. a. Young root; b. young leave; c. young stem; d. petal; e. calyx; f. stamen; g. young berry; h. coloring berry; i. mature berry; j. selection effect.


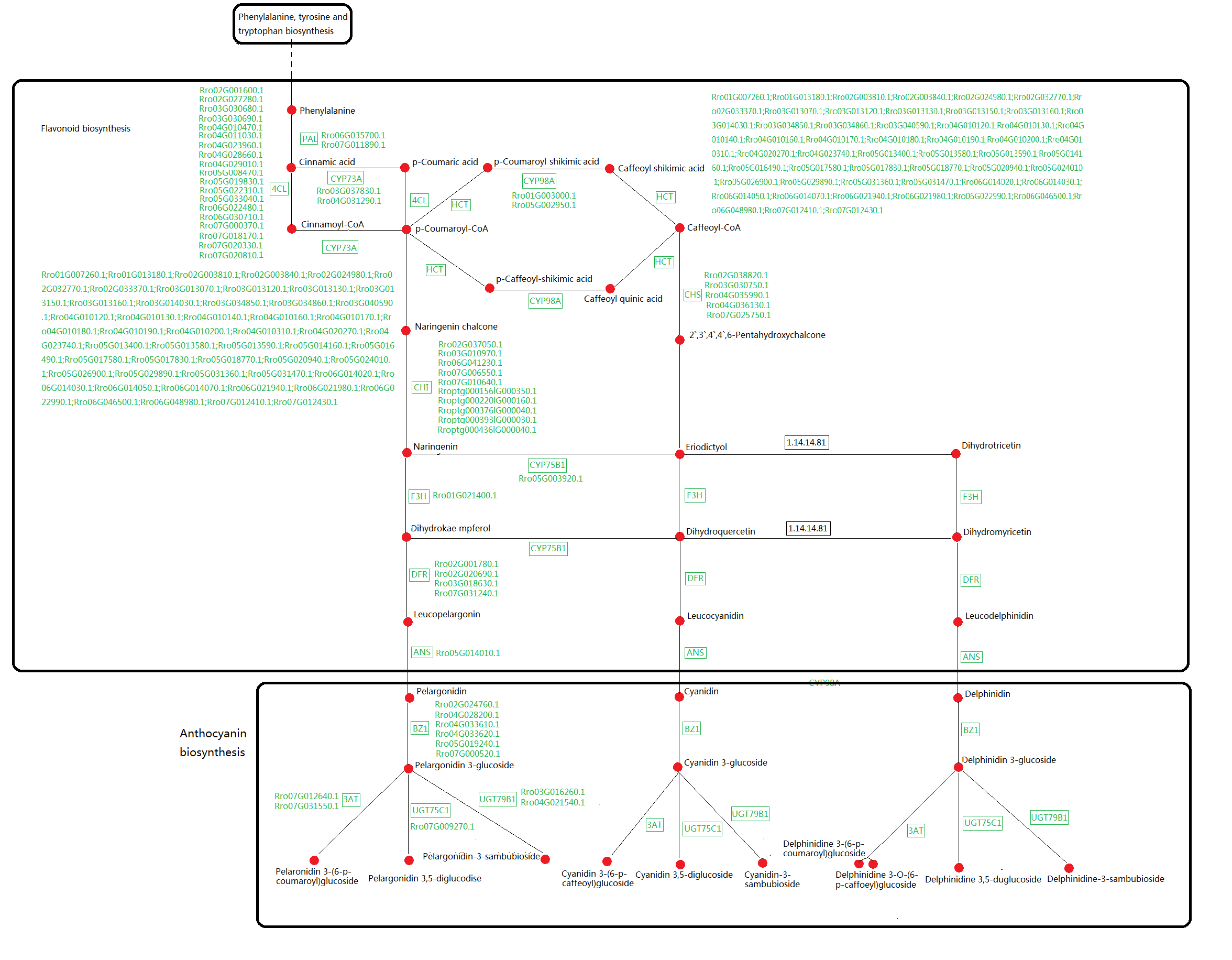


Fig. S10. Genes related to the biosynthesis of anthocyanin in *R. rosaefolius*, determined via KEGG annotation


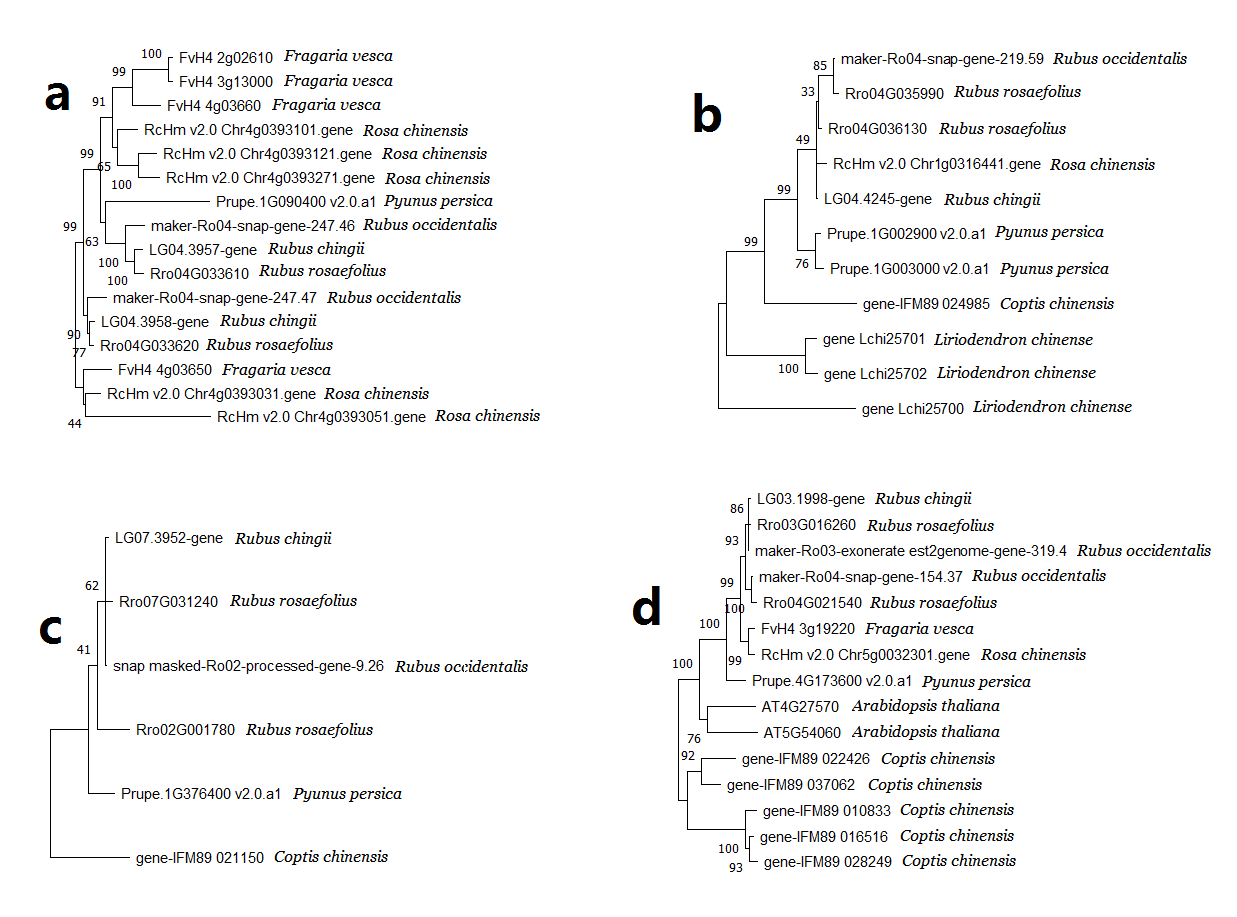


Fig. S11. Phylogenetic tree of the gene families. a. The OG0001492 family encodes BZ1; b. the OG0004740 family encodes CHS; c. the OG0015738 family encodes DRF; d. the OG0001588 family encodes 370 UGT79B1

Table S1. Statistical information on the short-read sequencing data used for K-mer analysis of the genome of *R. rosaefolius*

| Items | Counts |
| --- | --- |
| Library type | 350 bp |
| Data (Gb) | 59.76 |
| Depth (×) | 271.01 |
| Q20 (%) | 97.53 |
| Q30 (%) | 93.00 |

Table S2. Results of the genome property determination of *R. rosaefolius* by K-mer analysis

| Items | Counts |
| --- | --- |
| Genome size | 220.50 Mb |
| Repetitive ratio | 33.31% |
| Heterozygosity | 1.64% |
| GC content | 38.33% |

Table S3. Statistical information and summary of long reads used for assembly of the draft genome of *R. rosaefolius*

| Items | Counts |
| --- | --- |
| Long reads number | 768,242 |
| Long read bases | 13,041,734,377 |
| N50 of long reads | 17,013 |
| Average length of long reads | 16,976 |
| Max length of long reads | 40,422 |

Table S4. Assembly statistics of the primary draft genome of *Rubus rosaefolius* Smith at the contig level

| Items | Counts |
| --- | --- |
| Contig number | 141 |
| Contig length | 241,764,354 |
| N50 of contigs | 15,358,640 |
| N90 of contigs | 8,577,561 |
| Max length contig | 32,755,413 |
| GC content | 35.73 |

Table S5. Statistical information on the Hi-C sequencing data

| Items | Counts |
| --- | --- |
| Total Read Pairs | 165,307,537 |
| Base Number | 49,377,675,338 |
| N (%) | 0.00 |
| Q20 (%) | 97.87 |
| Q30 (%) | 94.22 |
| Mapped Reads | 274,945,079 |
| Unique Mapped Read Pairs | 122,506,571 |
| Unique Paired Alignments | 122,506,571 |
| Valid Interaction Pairs | 72,795,022 |
| Dangling End Pairs | 38493494 |
| Re-ligation Pairs | 1,939,943 |
| Self-cycle Pairs | 478,312 |
| Dumped Pairs | 8,799,800 |

Table S6. Draft genome corrected by Hi-C data

| Items | Counts |
| --- | --- |
| Contig number | 138 |
| Total contig length | 221,947,128 bp |
| N50 of contigs | 16,127,640 bp |
| Max contig | 32,755,413 bp |
| GC% of total contig | 35.82 |
| Scaffold number | 131 |
| N50 of scaffolds | 30,003,394 bp |
| Max scaffold | 36,279,055 bp |

Table S7. Statistical information on the Hi-C assembly

| Pseudochromosomes Code | Cluster Number | Cluster Length | Order Number | Order Length |
| --- | --- | --- | --- | --- |
| LG01 | 29 | 26,341,108 | 2 | 24,817,599 |
| LG02 | 2 | 36,278,955 | 2 | 36,278,955 |
| LG03 | 4 | 30,087,097 | 2 | 30,003,294 |
| LG04 | 23 | 34,257,668 | 2 | 33,334,161 |
| LG05 | 4 | 34,393,038 | 3 | 29,603,821 |
| LG06 | 4 | 30,216,138 | 2 | 30,095,508 |
| LG07 | 4 | 27,448,186 | 1 | 27,094,810 |
| Total (Ratio %) | 70 (50.72) | 219,022,190 (98.68) | 14 (20.00) | 211,228,148 (96.44) |

Table S8. Assembly integrity assessment of the contig-level draft genome of *R. rosaefolius* obtained by blasting against the cluster of essential genes (CEG) database

| Alignment Databases  (Reference alignment gene number) | Alignment Number | Alignment Ratio |
| --- | --- | --- |
| CEG* (458) | 456 | 99.56% |
| Highly conserved CEGs (248) | 241 | 97.18% |

Table S9. Assembly integrity assessment of the contig-level draft genome of *R. rosaefolius* obtained by blasting against the benchmarking universal single-copy ortholog database BUSCO v4.0

| Items | Counts (Percentage) |
| --- | --- |
| Total Lineage BUSCOs | 1,614 (100%) |
| Complete BUSCOs(C) | 1586 (98.27%) |
| Complete and single-copy BUSCOs(S) | 1550 (96.03%) |
| Complete and duplicated BUSCOs(D) | 36 (2.23%) |
| Fragmented BUSCOs(F) | 4 (0.25%) |
| Missing BUSCOs(M) | 24 (1.49%) |

Table S10. Statistical information on the assembly integrity and the uniformity of sequencing coverage obtained by reblasting the contig-level draft genome against short-read data

| Items | Counts |
| --- | --- |
| Total reads | 373,567,922 |
| Mapped reads | 333,533,326 |
| Mapped (%) | 89.28 |
| Properly mapped reads | 319,503,762 |
| Properly mapped (%) | 85.53 |
| Average depth | 220 X |
| Coverage | 99.76% |
| Coverage (≥5X) | 99.51% |
| Coverage (≥10X) | 99.29% |
| Coverage (≥20X) | 98.87% |

Table S11. Statistical information on the assembly integrity and the uniformity of sequencing coverage obtained by reblasting the contig-level draft genome against long-read data

| Items | Counts |
| --- | --- |
| Total reads | 768,242 |
| Mapped reads | 699,844 |
| Mapped (%) | 90.10 |
| Average depth | 44X |
| Coverage | 99.99% |
| Coverage (≥5X) | 99.62% |
| Coverage (≥10X) | 98.89% |
| Coverage (≥20X) | 94.04% |

Table 12. Statistical information on interspersed repeats in the draft genome

| Type | Number | Length | Rate (%) |
| --- | --- | --- | --- |
| ClassI:Retroelement | 77,214 | 38,632,841 | 17.41 |
| ClassI/DIRS | 4 | 268 | 0 |
| ClassI/LINE | 16,276 | 4,655,511 | 2.10 |
| ClassI/LTR/Caulimovirus | 549 | 673,578 | 0.3 |
| ClassI/LTR/Copia | 9,165 | 7,393,527 | 3.33 |
| ClassI/LTR/ERV | 3,468 | 236,251 | 0.11 |
| ClassI/LTR/Gypsy | 10,050 | 10,910,560 | 4.92 |
| ClassI/LTR/Ngaro | 414 | 99,241 | 0.04 |
| ClassI/LTR/Pao | 71 | 6,464 | 0 |
| ClassI/LTR/Unknown | 32,956 | 13,986,979 | 6.30 |
| ClassI/SINE | 4,261 | 670,462 | 0.3 |
| ClassII:DNA transposon | 69,195 | 17,752,910 | 8.00 |
| ClassII/Academ | 1 | 45 | 0 |
| ClassII/CACTA | 1,567 | 129,143 | 0.06 |
| ClassII/Crypton | 41 | 2,202 | 0 |
| ClassII/Dada | 207 | 10,728 | 0 |
| ClassII/Ginger | 27 | 993 | 0 |
| ClassII/Helitron | 575 | 348,439 | 0.16 |
| ClassII/IS3EU | 178 | 9,869 | 0 |
| ClassII/Kolobok | 201 | 13,210 | 0.01 |
| ClassII/Maverick | 50 | 2,233 | 0 |
| ClassII/Merlin | 187 | 9,880 | 0 |
| ClassII/Mutator | 499 | 51,050 | 0.02 |
| ClassII/P | 90 | 4,919 | 0 |
| ClassII/PIF-Harbinger | 1,496 | 492,663 | 0.22 |
| ClassII/PiggyBac | 42 | 1,629 | 0 |
| ClassII/Tc1-Mariner | 653 | 117,391 | 0.05 |
| ClassII/Unknown | 60,755 | 16,178,964 | 7.29 |
| ClassII/Zisupton | 48 | 2,274 | 0 |
| ClassII/hAT | 2,578 | 377,278 | 0.17 |
| Unknown | 20 | 1,021 | 0 |
| Total | 146,429 | 56,386,772 | 25.41 |

Table 13. Statistical information on tandem repeat sequences in the draft genome

| Type | Number | Length | Rate (%) |
| --- | --- | --- | --- |
| Microsatellite (1–9 bp units) | 112,543 | 4,706,682 | 2.12 |
| Minisatellite (10–99 bp units) | 33,969 | 15,279,422 | 6.88 |
| Satellite (>= 100 bp units) | 5,315 | 2,860,549 | 1.29 |
| Total | 151,827 | 22,846,653 | 10.29 |

Table 14. Statistical information on the prediction of protein-coding genes

| Method | Software | Species | Gene number |
| --- | --- | --- | --- |
| Ab initio | Augustus | - | 21,556 |
|  | SNAP | - | 34,854 |
| Homology-based | GeMoMa | *A. thaliana* | 20,759 |
|  |  | *F. vesca* | 26,369 |
|  |  | *R. chinensis* | 28,159 |
|  |  | *R. chingii* | 28,116 |
|  |  | *R. idaeus J* | 29,037 |
|  |  | *R. occidentalis* | 26,261 |
| RNAseq | GeneMarkS-T | - | 16,163 |
|  | ASA | - | 11,211 |
| Integration | EVM | - | 28,067 |

Table S15. Information on the predicted protein-coding genes of *R. rosaefolius* and six other species

| Species | Gene Number | Total Gene  Length | Average Gene  Length | Average Exon Length | Total Exon Number | Average Exon Number | Total Intron Length | Aveage Intron Length | Total Intron Number | Average Intron number |
| --- | --- | --- | --- | --- | --- | --- | --- | --- | --- | --- |
| *Rubus idaeus* | 33865 | 99490208 | 2937.85 | 1434.89 | 158058 | 4.67 | 50897612 | 1502.96 | 124193 | 3.67 |
| *Rubus rosaefolius* | 28067 | 85944749 | 3062.13 | 1483.90 | 144765 | 5.16 | 44296036 | 1578.22 | 116698 | 4.16 |
| *Rubus chingii* | 28877 | 80985278 | 2804.49 | 1096.81 | 135747 | 4.70 | 49312827 | 1707.69 | 106870 | 3.70 |
| *Rosa chinensis* | 45469 | 117351509 | 2580.91 | 1396.61 | 195894 | 4.31 | 53848932 | 1184.3 | 150425 | 3.31 |
| *Rubus occidentalis* | 33248 | 107185068 | 3223.80 | 1331.48 | 174731 | 5.26 | 62916137 | 1892.33 | 141483 | 4.26 |
| *Arabidopsis thaliana* | 27336 | 60260578 | 2204.44 | 1481.40 | 145214 | 5.31 | 19765115 | 723.04 | 117878 | 4.31 |
| *Fragaria vesca* | 33998 | 100436889 | 2954.20 | 1558.36 | 164419 | 4.84 | 47455847 | 1395.84 | 130421 | 3.84 |

Table S16. Completeness of the predicted protein-coding genes

| Items | Count (Ratio) |
| --- | --- |
| Complete BUSCOs(C) | 1555 (96.34%) |
| Complete and single-copy BUSCOs(S) | 1519 (94.11%) |
| Complete and duplicated BUSCOs(D) | 36 (2.23%) |
| Fragmented BUSCOs(F) | 29 (1.80%) |
| Missing BUSCOs(M) | 30 (1.86%) |
| Total Lineage BUSCOs | 1614 (100%) |

Table S17. Information on the annotations of the predicted protein-coding genes

| Annotation Database | Annotated Number | Annotated Ratio |
| --- | --- | --- |
| GO | 22,011 | 78.42 |
| KEGG | 19,516 | 69.53 |
| KOG | 14,078 | 50.16 |
| Pfam | 22,448 | 79.98 |
| Swissprot | 20,579 | 73.32 |
| TrEMBL | 26,024 | 92.72 |
| eggNOG | 21,436 | 76.37 |
| Nr | 25,503 | 90.86 |
| All | 26,173 | 93.25 |

Table S18. Statistical information on the predicted RNA genes and pseudogenes

| Items | Counts |
| --- | --- |
| rRNA | 851 |
| tRNA | 710 |
| miRNA | 54 |
| snRNA | 78 |
| snoRNA | 95 |
| pseudogene | 51 |

Table S19. Statistical information on the gene families of 10 genomes

| Items | *Arabidopsis thaliana* | *Coptis chinensis* | *Fragaria vesca* | *Liriodendron chinens*e | *Oryze sativa* | *Pyunus persica* | *Rosa chinensis* | *Rubus chingii* | *Rubus occidentalis* | *Rubus rosaefolius* |
| --- | --- | --- | --- | --- | --- | --- | --- | --- | --- | --- |
| Gene number | 27,316 | 36,637 | 33,915 | 33,181 | 24,343 | 26,486 | 45,464 | 27,288 | 29,137 | 28,067 |
| Genes number in orthogroups | 18,088 | 26,452 | 27,517 | 27,865 | 13,634 | 22,933 | 34,007 | 23,719 | 22,862 | 25,828 |
| Unassigned genes number | 9,228 | 10,185 | 6,398 | 5,316 | 10,709 | 3,553 | 11,457 | 3,569 | 6,275 | 2,239 |
| Orthogroups number containing species | 12,403 | 15,283 | 19,464 | 13,610 | 9,585 | 17,456 | 21,570 | 17,576 | 17,970 | 19,547 |
| Number of species-specific orthogroups | 2,448 | 2,289 | 831 | 1,242 | 2,120 | 317 | 1,600 | 255 | 626 | 159 |
| Number of genes in species-specific orthogroups | 6,596 | 7,871 | 2,658 | 9,875 | 5,294 | 1,026 | 4,685 | 682 | 1,738 | 702 |

Note: The genes containing frameshift mutations in all species are excluded from the statistics.

Table S20. Significant differences in expression between duplicated gene pairs that occurred due to large genome duplication events

| Tissues | Informative pairs | Number of significant difference pairs | Ratio of significant difference pairs (%) | Number of non-Significant difference pair | Ratio of non-significant difference pairs (%) |
| --- | --- | --- | --- | --- | --- |
| Young root | 2663 | 2035 | 76.42 | 628 | 23.58 |
| Petal | 2771 | 1997 | 72.07 | 714 | 27.93 |
| Calyx | 2699 | 1957 | 72.51 | 742 | 27.49 |
| Stamen | 2917 | 2245 | 76.96 | 672 | 23.04 |
| Yung stem | 2709 | 1989 | 73.42 | 720 | 26.58 |
| Young berry | 2753 | 1985 | 72.10 | 768 | 27.90 |
| Coloring berry | 2760 | 1960 | 71.01 | 800 | 28.99 |
| Mature berry | 2567 | 1935 | 75.38 | 632 | 24.62 |
| Young leaf | 2836 | 2064 | 72.27 | 772 | 27.73 |
